# Perceptions and Experiences of Animal‐Assisted Interventions for People Living With Dementia: A Qualitative Evidence Synthesis

**DOI:** 10.1111/jocn.17429

**Published:** 2024-10-06

**Authors:** Dou Zhang, Marita Hennessy, Qiuxia Li, Nuala Paley, Gerry Paley, Catherine Houghton

**Affiliations:** ^1^ School of Nursing and Midwifery University of Galway Galway Ireland; ^2^ College of Medicine and Health University College Cork Cork Ireland; ^3^ School of Health and Sciences, University of Galway Galway Ireland; ^4^ The Dementia Research Advisory Team, The Alzheimer Society of Ireland Dublin Ireland

## Abstract

**Aim:**

To synthesise stakeholders' experiences and perceptions of animal‐assisted intervention (AAI) for people with dementia in community care settings.

**Design:**

Qualitative evidence synthesis.

**Methods:**

We systematically searched Medline, CINAHL, Embase, Scopus, Web of Science, PsycINFO and AgeLine for potentially eligible studies. Thematic synthesis was used to analyse the data from included studies. We assessed the methodological limitations of included studies using an adaptation of the Critical Appraisal Skills Programme checklist and used Confidence in the Evidence from Reviews of Qualitative Research (GRADE‐CERQual) tool to assess confidence in review findings. This review is reported using the ENTREQ checklist.

**Results:**

We included 14 reports from 11 studies and developed three analytical themes incorporating a gardening analogy: planting–connecting with animals, growing–engaging in AAI and nurturing–making AAI work; and six subthemes: willingness to connect, building relationships, a rich experience, the benefits of AAI, individualised and holistic approach and training and support, with 15 key findings.

**Conclusion:**

This review describes people's experiences and perceptions of AAI for people with dementia, and provides recommendations on the development and implementation of AAI, with moderate to high confidence. Nurses need to consider the factors that influence the implementation of AAI identified in this review, to facilitate engagement and long‐term impacts while adopting AAI in community care settings.

**Implications for the Profession and Patient Care:**

This review may enhance healthcare professionals' understanding of AAI for people with dementia in community care settings. AAI is a complex intervention that can be delivered in varied manner. A multicomponent, flexible and individualised AAI is important. Additional training and education for staff are needed.

**Patient or Public Contribution:**

A man with dementia and his wife who share a love of dogs, advised at each step of the review, providing insights and perspectives and contributing as co‐authors.

**Summary**

- This review highlights people with dementia, care partners and service providers perceived very positive experiences, interactions and benefits throughout AAI in community care settings.
- AAI is a complex intervention that can be delivered in varied manner: Individualised and multicomponent AAI tailored to people's needs and preferences are facilitators of engagement.
- There is a further need for additional training, materials and policies guiding AAI in community care settings to ensure a safe and effective service delivery for people with dementia.

## 1 Introduction

Dementia is a priority public health issue, with rapidly rising rates of new cases; but yet without effective treatments to prevent or slow the progress and cure the disease (Rogan et al. 2023; World Health Organisation 2017). More than 90% of people living with dementia are affected by behavioural and psychological symptoms of dementia (BPSD), such as agitation, depression and anxiety (Bessey and Walaszek 2019), which are mostly attributed to people's unmet needs (Cohen‐Mansfield 2018). As BPSD has a negative impact on people with dementia and their caregivers, the World Health Organisation (WHO) indicates that it is significantly important to provide support addressing their needs and to improve their quality of life (QoL) and well‐being (WHO 2017).

Moreover, discovering and developing diverse therapeutic approaches is an identified dementia research priority (Rogan et al. 2023; Shah et al. 2016). Currently, nonpharmacological approaches are advocated as essential and alternative first‐line treatments to reduce BPSD and alleviate cognitive decline (e.g., arts therapy and music therapy) (Alzheimer Disease International 2022; Dyer et al. 2016; Leng, Zhao, and Wang 2020; Tible et al. 2017). Animal‐assisted intervention (AAI) is a popular type of psychosocial and nonpharmacological intervention (Fondation Médéric Alzheimer 2024; Chang et al. 2021; Leng, Zhao, and Wang 2020), which is potentially effective in improving health and well‐being of people with dementia (Forget et al. 2021; Marks and McVilly 2020; Yakimicki et al. 2019). AAI is increasingly being used in community care settings (Schuurmans et al. 2016). The terminology of community care settings can vary internationally (Heller 2002). In this review, we define it as ‘community‐based care facilities offering health and social care services and support for residents in need’ (National Institute on Aging 2024; Orellana, Manthorpe, and Tinker 2020; Mijnarends et al. 2015; Toth et al. 2022), AAI in personal homes and acute hospital settings were excluded.

## 2 Background

The International Association of Human‐Animal Interaction Organisations (IAHAIO) defines AAI as structured and goal‐oriented psychosocial interventions by trained human–animal teams for therapeutic gains in health, education and human services (Fondation Médéric Alzheimer 2024; IAHAIO 2018). AAI can be carried by diverse health and social care professionals (e.g., physicians, therapists and nurses) (Fondation Médéric Alzheimer 2024; Morrison 2007). As such nurses play a key role in closely working with residents and multidisciplinary coordinators for AAI and providing quality care services (Buettner, Fitzsimmons, and Barba 2011; Fine 2019).

For people with dementia, AAI can include a variety of animals, for example, dogs, horses, sheep, goats, cats, chickens, rabbits and fish (Babka, Lane, and Johnson 2021; Hu et al. 2018); with some studies using robotic pets as a substitute (Lai et al. 2019; Shoesmith, Surr, and Ratschen 2023). Delivery of AAI for people with dementia varies through scheduled activities, such as petting, grooming, talking, walking and playing, with goals of enhancing interactions and cognitive stimulation (Batubara et al. 2022; Chen et al. 2022; Klimova, Toman, and Kuca 2019). Such human–animal interactions and sensory experiences with animals can stimulate motivation and positive feelings and potentially have positive impacts on a range of outcomes, including BPSD (Chen et al. 2022; Hu et al. 2018), social behaviours (Klimova, Toman, and Kuca 2019), physical activity, dietary intake and QoL (Marks and McVilly 2020; Shoesmith, Surr, and Ratschen 2023; Yakimicki et al. 2019).

While AAI shows encouraging results for people with dementia, the evidence is not yet conclusive. A systematic review by Lai et al. (2019) compared the efficacy of AAI among live animals, robotic animals and soft toys for people with dementia. They found that while AAI could slightly reduce depression, it had no effect on QoL, cognitive function or daily activities among people with dementia. Similarly, more recent systematic reviews of randomised trials and cohort studies (Batubara et al. 2022; Chen et al. 2022) also found mixed effects of AAI. Review authors highlight that inconsistent and inconclusive results were mainly due to insufficient data, limited sample sizes and low quality of the primary studies (Lai et al. 2019; Marks and McVilly 2020). This emphasises the need for further evidence of the impact and value of AAI.

To date, research on AAI for people with dementia has been primarily quantitative, although some qualitative studies have been included in mixed‐method systematic reviews (Orr et al. 2023; Shoesmith, Surr, and Ratschen 2023). However, these were not synthesised exclusively. One mixed‐method systematic review (Orr et al. 2023) examined the impacts of AAI on health and well‐being for institutionalised older adults (not specifically for people with dementia), using personal pets (which do not strictly meet the criteria for AAI), residential animals and visiting animals. Another mixed‐method systematic review by Shoesmith, Surr, and Ratschen (2023) compared AAI and robotic animal intervention for people with dementia. However, this review focused on the characteristics and differences between AAI and robotic animal interventions and drew few insights into people's experiences and attitudes of how well AAI was implemented. The authors, in both reviews, also expressed difficulty in comparing different types of AAI due to the variability of delivery approach (Orr et al. 2023; Shoesmith, Surr, and Ratschen 2023).

Therefore, a qualitative evidence synthesis of AAI specifically for people with dementia is needed to address this evidence gap. Qualitative syntheses can help answer the questions beyond systematic reviews of quantitative research on ‘what works’ and can help to understand the complex intervention of implementation and its impacts on different subgroups of people, and in different contexts (Flemming and Noyes 2021). Our review question explores stakeholders' perceptions and experiences of AAI for people with dementia in community care settings and the potential factors influencing its implementation. This would significantly contribute to the evidence base of AAI, guideline development and clinical implications for people with dementia and healthcare professionals.

### 2.1 Objectives

The aim of this review was to explore stakeholders' perceptions and experiences of AAI for people with dementia in community care settings. The specific objectives were to:

- Explore stakeholders' perceptions and experiences of implementing AAI for people with dementia.
- Identify the factors influencing the delivery of AAI for people with dementia.
- Examine any differences in the above, by types of animals, settings and stages of dementia.
- Develop recommendations to enhance the implementation and impact of AAI for people with dementia.

## 3 Methods

The review is reported following the guidance of Cochrane Qualitative Evidence Synthesis: Protocol and Review Template, Version 1.4b (Glenton et al. 2023), and Enhancing Transparency in Reporting the Synthesis of Qualitative Research: ENTREQ checklist (Tong et al. 2012), see File S1. The review protocol (Zhang, Hennessy, and Houghton 2023) was preregistered on the International Prospective Register of Systematic Reviews (PROSPERO) database (No. CRD42023393938).

### 3.1 Criteria for Considering Studies for This Review

#### 3.1.1 Types of Studies

Primary studies that employed qualitative study designs such as ethnography, phenomenology, case studies, grounded theory studies, action research and qualitative process evaluations were included. Mixed‐method studies including qualitative data using qualitative data collection and analysis methods, from which the qualitative data could be extracted, were included. No filters for publication date, language and country were applied.

#### 3.1.2 Topic of Interest

The SPICE (setting, perspective, intervention, comparison and evaluation) tool (Booth 2006) was used to formulate the research question and search strategy (File S2). Studies focusing on stakeholders' experiences and perceptions of AAI for people with dementia were included. We adopted the IAHAIO (2018) definition of AAI. We only included face‐to‐face AAI, with all types of live animals; we excluded AAI with robotic pets, animal‐shaped soft toys and personal pets.

Stakeholders included people with dementia and their family members, healthcare professionals and animal handlers. People with dementia at any age or stage, and their care partners were eligible for inclusion. Healthcare professionals referred to staff of residential care/nursing homes caring for people with dementia, such as nurses. Paid or unpaid staff working in/with animal organisations or therapy centres (e.g., animal handlers, AAI facilitators and volunteers) were included.

Studies from community care settings were included, for example, nursing homes, care homes, assisted living facilities, continuing care communities, day care centres and rehabilitative centres (e.g., therapy centres/care farms). Studies of AAI based in hospitals and personal homes were excluded. Studies focusing on AAI or animals without people with dementia were excluded.

### 3.2 Search Methods for Identification of Studies

The search strategy was developed in collaboration with an experienced information specialist.

#### 3.2.1 Electronic Searches

We systematically searched the following electronic databases in February 2023: Medline (Ovid), CINAHL (EBSCOhost), Embase (Ovid), Scopus (Elsevier), Web of Science core collection (Clarivate), PsycINFO (EBSCOhost) and AgeLine (EBSCOhost). The search strategy was developed in Medline and adapted for other databases. See File S3 for details.

#### 3.2.2 Searching Other Resources

In addition to searching the databases outlined, we searched ProQuest Dissertations & Theses A&I and PsycEXTRA. We conducted reference and citation checking of included articles via handsearching Web of Science and Google Scholar, respectively, and contacted related author(s) to source full texts and/or to clarify any missing information, where necessary (Booth 2016).

### 3.3 Selection of Studies

**FIGURE 1:**
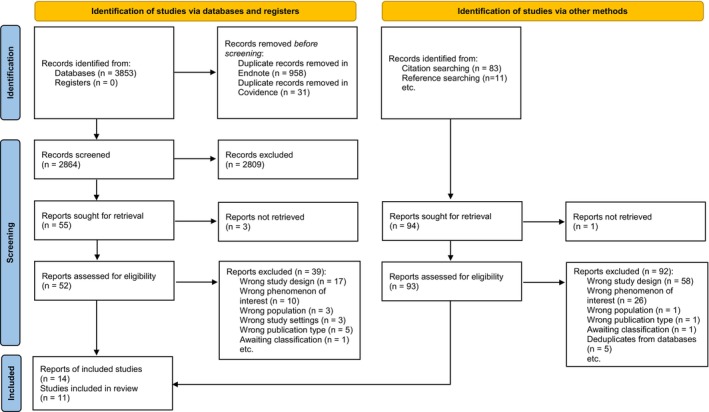
PRISMA flow diagram. [Colour figure can be viewed at wileyonlinelibrary.com]

Retrieved records were imported into Endnote 20 (Clarivate) for deduplication. Remaining records were exported to Covidence and screened independently by two reviewers DZ and QL (Harrison et al. 2020). Titles and abstracts of identified records, and then potentially eligible full texts, were screened against the eligibility criteria. Titles and abstracts not published in English were initially screened using Google Translate. Any disagreements between reviewers (DZ and QL) were resolved through discussion with a third reviewer (CH or MH). We used the PRISMA 2020 flow diagram to record the selection process (Figure 1). Two studies (Schär 2015; Tribet, Boucharlat, and Myslinski 2007) were potentially eligible for inclusion after title and abstract screening, but we were unable to translate the full texts into English due to resource constraints. Therefore, they were listed as ‘studies awaiting classification’, as recommended by EPOC (Glenton et al. 2023).

### 3.4 Data Extraction

A specifically designed data extraction form was used to capture contextual and methodological information, including first author, year, country, purposes, research design, study setting, type of participant, type of intervention, sample size, data collection and analysis methods, and theory or models of the studies. We extracted all data relevant to the review objectives, including themes, categories and quotes, into QSR NVivo. Data extraction was piloted, completed and checked by reviewers (DZ and QL) (Table 1).

**TABLE 1:** Characteristics of the included studies.

Author (year)	Country	Study setting	Study aims	Study design	Study participants	Intervention description	Theory or models
**Study 1:** Busselman (2017)	United States	A therapeutic riding centre and a memory care home	To describe positive and negative quality‐of‐life indicators and environmental correlates, demonstrated by people with dementia who participated in Riding in the Moment	Mixed‐methods case study Purposively maximum variation sampling; qualitatively describing field notes data	*N* = 4 People living with dementia (two males, two females, age ranged 71 to 95, dementia stage ranged from early–moderate to late–moderate stage)	Horses Riding in the Moment programme, include five main occupational opportunities: riding, grooming and petting a horse, putting on or taking off helmets or coats and bench time; empowering participants to choose activities and tailor to people's special needs; 1 h session, once a week and 4 weeks	The Lived Environment Life Quality Model
**Study 2:** Casey et al. (2018)	Canada	A specialised dementia unit in an LTC home	To examine if changes among residents with dementia living in a long‐term care facility by AAI had any affect on the work environment and the staff	Qualitative study Sampling: NS Thematic analysis of qualitative in‐depth interviews collected by 8‐item open‐ended questionnaire	*N* = 20 Staff work in the specialised dementia unit (two males, 18 females, ages ranged from early 20s to mid‐50s, personal service workers, registered nurses, dietary aids, housekeepers and recreation therapists)	A sheep, rabbits, chickens and a goat Petting, grooming and walking the animals; singing animal songs, learning about the animal's history, species and breed, training the animal; reminiscing about past human–animal interactions and relationships, and reflecting on the animal's behaviours and story in relation to their own story; three 1 h sessions, once a week, 8 weeks	NS
**Study 3:** Fields (2017), Fields, Wood, and Lassell (2019)	United States	A for‐profit LTC facility and a nonprofit therapeutic riding centre	To investigate the influence and acceptability of the equine‐assisted intervention on the quality of life for adults with dementia residing in an institutional home	Mixed‐methods case study Convenience and purposive sampling Basic qualitative description of interviews and filed notes data	*N* = 11 Six people living with dementia (four females, two males, ages ranged 71–95, mild‐to‐late moderate stage); and five service providers (worked in LTC and riding centre)	Three horses and a pony Various groundwork activities such as grooming, petting, riding the horse, social interactions on bench; embedding dementia care in AAI, recognising participants' needs and interests currently; 1 h session, once a week, 8 weeks	Lived environment life quality (LELQ) model
**Study 4:** Hodgson et al. (2022)	United Kingdom	Four care homes	To assess the impacts of HenPower on people living with dementia in care homes and care home staff's views and experiences of HenPower	Qualitative study Purposive sampling Thematic analysis of interviews and observations data	*N* = 54 29 people living with dementia (23 females, six males) and 25 care home staff	Hens Hen‐keeping and hen‐related creative activities (designing and making hen houses, singing, ceramics and poetry about hens), led by a trained artist; providing both one‐to‐one and group activities to achieve active engagement in individualised ways; sustained programme	Theory of change model
**Study 5:** Kawamura, Niiyama, and Niiyama (2009)	Japan	A private nursing home	To determine institutionalised elderly Japanese women's perceptions of animal‐assisted activity and how it may be relevant to clinical nursing practices	Qualitative study Sampling: NS Interviews were analysed using Colaizzi's phenomenological methodology	*N* = 8 People living with dementia (all female, age ranged from 67 to 94, dementia stage ranged very mild to mild)	Four dogs Freely feed, hold and play with the dogs, each dog was placed on a separate table and the participants watched or played with the dogs for approximately 30 min; 2 h session, twice a month, 2 years	NS
**Study 6:** Kongable, Stolley, and Buckwalter (1990)	United States	A special care Alzheimer unit in a home for veterans	To explore nursing staff members' view of benefits, risks, behavioural responses and significant changes in using a therapy dog programme in a special care AD unit	Qualitative study Random basis sampling and purposive sampling Content analysis of interviews with 5 open‐ended questions	*N* = 6 Nursing staff (director of nursing, the coordinator of the pet therapy programme, registered nurses and nursing assistants)	Dog Activities: NS, it became permanent activity eventually; 3 h session, once a week	NS
**Study 7:** Lassell, Fields, et al. (2022)	United States	A therapeutic horseback riding centre	To explore the appropriateness of an adaptive riding programme for people living with dementia through examining family members' (care partners) reported outcomes	Case study Convenience sampling Theoretical thematic analysis of field notes and interview data	*N* = 10 Five people living with dementia (Four females and a male, mean age was 70, dementia stage ranged mild‐to‐moderate–severe); and five care partners (three spouses and two children, two males and three females, mean age was 58)	Horse and donkey Adaptive riding programme providing various activities with horses, e.g., grooming, petting, riding, observing; available to individuals and groups with diverse capacities and needs; 1 h session, once a week, 8 weeks	The Positive emotions, Engagement, Relationships, Meaning, Accomplishment (PERMA) theory of well‐being
**Study 8:** Lassell, Cross, et al. (2022)	United States	A therapeutic riding centre and a senior centre	To explore the feasibility and acceptability of an adaptive riding programme with dyads (people living with dementia, family care partners) and a gardening comparison condition	Mixed methods feasibility study Sampling: NS Thematic analysis of interviews and field notes data	*N* = 10 Five people living with dementia (age ranged 57 to 84, dementia type varied from early onset Alzheimer disease to vascular); and five family care partners (three spouses and two daughters, three females, two males age ranged 37 to 72)	Horses, pony or donkeys Adaptive riding programme involved grooming (20 min) riding (15–20 min), petting (5–10 min) and transitions and unstructured time/observing ongoing activities (5–10 min), providing personalised conversation cards; tailoring to participants' needs; 1 h session, once a week, 8 weeks	NS
**Study 9:** Swall (2015), Swall et al. (2015, 2017)	Sweden	A municipal nursing home	To illuminate the meaning of care for people with AD and the lived experience of encounters with a therapy dog for persons with AD	Qualitative study Convenience and snowball sampling Phenomenological hermeneutical method of observation data	*N* = 5 People living with dementia (four females, one male, ages ranged from 89 to 95, medium‐to‐severe AD)	Dog Individualised visit and differed according to the circumstances of person. Activities included close contact, talking, cuddling and playing (e.g., throwing balls, searching for hidden sweets) with the dog, which took place in a specially adapted room, equipped with toys, blankets, pictures of dogs, chairs, tables and a couch; 2.5 h session, once a week, 10 weeks	NS
**Study 10:** Swall (2015), Swall et al. (2016)	Sweden	Eight municipal metropolitan nursing homes	To illuminate meanings of the lived experiences of dog handlers' when visiting older persons with dementia with their therapy dog	Qualitative study Convenience and snowball sampling Phenomenon logical hermeneutical method analysis of interviews with 2 open‐ended questions	*N* = 9 Dog handlers (all females, registered nurses, occupational therapy assistants and assistant nurses)	Dog Individualised approach provided by dog handler based on participants' specific needs; including close contact with the dog by touching its fur, cuddling and talking, searching for hidden sweets or throwing balls, or other activities that may vary for each visit, depending on individual's condition, in a room specially adapted with toys for dogs, and pictures of dogs; 2.5 h session, once a week, 10 weeks	NS
**Study 11:** Swall et al. (2019)	Sweden	Seven municipal nursing home	To describe the experience of dog handler's visits to persons with dementia nearing the end of life	Qualitative study Convenience sampling Content analysis of interviews	*N* = 11 Dog handlers (all women, aged ranged 43 and 65; registered nurses, occupational therapy assistants and caregivers)	Dog Activities: NS Frequency and duration: NS	NS

Abbreviations: AAI, animal‐assisted intervention; AD, Alzheimer's disease; LTC, long‐term care facility; NS, not stated; RM, Riding in the Moment programme.

### 3.5 Assessing the Methodological Limitations of Included Studies

Methodological limitations of included studies were assessed by three independent reviewers (DZ, MH, CH), using an adapted Critical Appraisal Skills Program (CASP) qualitative studies checklist. This checklist is based on CASP (2018), and has since been revised and used in previous Cochrane Reviews (Ames et al. 2019; Glenton et al. 2021; Houghton et al. 2021). Disagreements were resolved through discussion among the three authors. We did not exclude studies based on quality assessment. Three studies were assessed as having ‘no or very minor concerns’, six studies were assessed as having ‘minor concerns’, one study was assessed as having ‘moderate concerns’ and one study was assessed as having ‘serious concerns’. We found many of the included studies lacked reporting of researcher reflexivity, some poorly reported ethical concerns and a few lacked details on sampling strategy, data collection and data analysis (Table 2).

**TABLE 2:** Assessment of methodological limitations.

Study ID	1. Were the settings and context described adequately?	2. Was the sampling strategy described, and was this appropriate?	3. Was the data collection strategy described and justified?	4. Was the data analysis described, and was this appropriate?	5. Were the claims made/findings supported by sufficient evidence?	6. Was there evidence of reflexivity?	7. Did the study demonstrate sensitivity to ethical concerns?	8. Any other concerns?	Overall assessment of methodological limitations
**Study 1:** Busselman (2017)	Yes	Yes	Yes	Partial	Yes	Yes	Yes	No	Minor concerns
**Study 2:** Casey et al. (2018)	Yes	Partial	Yes	Partial	Yes	Partial	Partial	No	Moderate concerns
**Study 3:** Fields (2017), Fields, Wood, and Lassell (2019)	Yes	Yes	Yes	Yes	Yes	Yes	Yes	No	Minor concerns
**Study 4:** Hodgson et al. (2022)	Yes	Yes	Partial	Yes	Yes	No	Yes	No	Minor concerns
**Study 5:** Kawamura, Niiyama, and Niiyama (2009)	Yes	Yes	Yes	Yes	Partial	No	Yes	No	Minor concerns
**Study 6:** Kongable, Stolley, and Buckwalter (1990)	Yes	Yes	Partial	Partial	Yes	No	No	Yes (study did not claim qualitative design)	Serious concerns
**Study 7:** Lassell, Fields, et al. (2022)	Yes	Yes	Yes	Yes	Yes	Yes	Partial	No	Minor concerns
**Study 8:** Lassell, Cross, et al. (2022)	Yes	Partial	Yes	Yes	Yes	Yes	Partial	No	Minor concerns
**Study 9:** Swall (2015), Swall et al. (2015, 2017)	Yes	Yes	Yes	Yes	Yes	Yes	Yes	No	No or very minor concerns
**Study 10:** Swall (2015), Swall et al. (2016)	Yes	Yes	Yes	Yes	Yes	Yes	Yes	No	No or very minor concerns
**Study 11:** Swall et al. (2019)	Yes	Yes	Yes	Yes	Yes	Partial	Partial	No	No or very minor concerns

### 3.6 Data Management, Analysis and Synthesis

QSR NVivo 2020 was used for data management and analysis. Our approach to the analysis was guided by the RETREAT framework (Booth et al. 2018). We identified thematic synthesis as an appropriate method for data analysis in this review (Thomas and Harden 2008). Thematic synthesis is a frequently used flexible approach to synthesise the findings from multiple primary qualitative studies and has the advantage of employing both ‘thin’ or ‘thick’ data to develop policy and guidance, and inform clinical practice and implementation (Flemming and Noyes 2021; Thomas and Harden 2008). This was deemed appropriate for informing decision‐making about AAI for people with dementia in community care settings.

Thematic synthesis comprises three stages: ‘line‐by‐line’ coding, developing the ‘descriptive themes’ and generating the ‘analytical themes’. Initially, the lead reviewer (DZ) conducted line‐by‐line coding of primary data; and then organised the free codes to generate descriptive themes, which generalised the description of experiences, impacts, factors and recommendations of AAI. Analytical themes were then generated through interpreting and explaining the findings beyond primary studies guided by the review question, which provided deeper understanding of the nature of AAI (Thomas and Harden 2008). Finally, a gardening analogy was developed to present and visualise the analytical themes. The analysis of above three steps was piloted, discussed and refined with other reviewers (CH and MH).

### 3.7 Assessing our Confidence in the Review Findings

Three independent review authors (DZ, MH, CH) used the ‘Confidence in the Evidence from Reviews of Qualitative research (GRADE‐CERQual)’ tool (Lewin et al. 2018) to assess the confidence of each key finding based on four components: methodological limitations, coherence, adequacy and relevance of data; rated as high, moderate, low and very low confidence. Of the 15 findings from the review, we graded 10 as ‘high confidence’ and 5 as ‘moderate confidence’. See summary of qualitative findings (Table 3) and the detailed results of GRADE‐CERQual (File S4).

**TABLE 3:** Summary of qualitative findings (SoQF).

Summary of review finding	Reports of studies contributing to the review finding	CERQual assessment of confidence in the evidence	Explanation of CERQual assessment
**Theme 1: Planting: Connecting with animals**			
*Subtheme 1: Willingness to connect*			
Finding 1: The willingness of people with dementia, care partners and staff to engage with AAI may depend on prior experience or their attitude towards animals in general	Casey et al. (2018), Fields (2017), Fields, Wood, and Lassell (2019), Hodgson et al. (2022), Kawamura, Niiyama, and Niiyama (2009), Kongable, Stolley, and Buckwalter (1990), Lassell, Cross, et al. (2022), Lassell, Fields, et al. (2022), Swall et al. (2016)	High confidence	No or very minor concerns regarding coherence and adequacy, and minor concerns regarding methodological limitations and relevance
Finding 2: Despite initial resistance, people with dementia may begin to engage with AAI after a few activities	Fields (2017), Hodgson et al. (2022)	Moderate confidence	No or very minor concerns regarding coherence, minor concerns regarding methodological limitations and moderate concerns regarding relevance and adequacy
*Subtheme 2: Building relationships*			
Finding 3: The animal, through intuition or training, may facilitate a therapeutic relationship with people with dementia	Fields (2017), Fields, Wood, and Lassell (2019), Kongable, Stolley, and Buckwalter (1990), Lassell, Fields, et al. (2022), Swall et al. (2015, 2017)	Moderate confidence	No or very minor concerns regarding coherence, minor concerns regarding adequacy and moderate concerns regarding methodological limitations and relevance
Finding 4: People with dementia may begin to feel a sense of protectiveness and responsibility for caring for the animals	Kawamura, Niiyama, and Niiyama (2009), Hodgson et al. (2022), Swall (2015), Swall et al. (2015, 2017)	Moderate confidence	No or very minor concerns regarding coherence, minor concerns regarding methodological limitations and adequacy and moderate concerns regarding relevance
Finding 5: Animals could be a catalyst for developing enhanced relationships between people with dementia and others through shared experiences with the animals	Fields (2017), Fields, Wood, and Lassell (2019), Hodgson et al. (2022), Kawamura, Niiyama, and Niiyama (2009), Lassell, Cross, et al. (2022), Kongable, Stolley, and Buckwalter (1990), Swall et al. (2016)	High confidence	Minor concerns regarding methodological limitations, coherence, relevance and adequacy
**Theme 2: Growing: Engaging in AAI**			
*Subtheme 1: A rich experience*			
Finding 6: Interactions in AAI could be a multidimensional experience from sensory input to physical and cognitive interactivity	Busselman (2017), Hodgson et al. (2022), Fields (2017), Fields, Wood, and Lassell (2019), Kongable, Stolley, and Buckwalter (1990), Lassell, Cross, et al. (2022), Lassell, Fields, et al. (2022), Swall (2015), Swall et al. (2015, 2016, 2017, 2019)	High confidence	No or very minor concerns regarding coherence and adequacy, and minor concerns regarding methodological limitations and relevance
Finding 7: Interaction with animals could trigger memories, positive and negative, for people with dementia	Fields (2017), Fields, Wood, and Lassell (2019), Kongable, Stolley, and Buckwalter (1990), Kawamura, Niiyama, and Niiyama (2009), Lassell, Fields, et al. (2022), Swall (2015), Swall et al. (2015, 2016, 2017)	High confidence	No or very minor concerns regarding coherence and adequacy, and minor concerns regarding methodological limitations and relevance
Finding 8: AAI provides opportunity for people with dementia to interact with others more socially and to connect with nature	Casey et al. (2018), Fields (2017), Fields, Wood, and Lassell (2019), Hodgson et al. (2022), Kongable, Stolley, and Buckwalter (1990), Kawamura, Niiyama, and Niiyama (2009), Lassell, Cross, et al. (2022), Lassell, Fields, et al. (2022), Swall (2015), Swall et al. (2015, 2016, 2017, 2019)	High confidence	No or very minor concerns regarding coherence and adequacy and minor concerns regarding methodological limitations and relevance
*Subtheme 2: The benefits of AAI*			
Finding 9: People with dementia, engaging AAI, may experience improved mood and physical improvements	Casey et al. (2018), Fields (2017), Fields, Wood, and Lassell (2019), Hodgson et al. (2022), Kongable, Stolley, and Buckwalter (1990), Kawamura, Niiyama, and Niiyama (2009), Lassell, Cross, et al. (2022), Lassell, Fields, et al. (2022), Swall (2015), Swall et al. (2015, 2016, 2017, 2019)	High confidence	No or very minor concerns regarding adequacy, and minor concerns regarding methodological limitations, coherence and relevance
Finding 10: AAI for people with dementia may have positive effects on cognitive functions and social ability, and may reduce BPSD symptoms	Casey et al. (2018), Fields (2017), Fields, Wood, and Lassell (2019), Hodgson et al. (2022), Kongable, Stolley, and Buckwalter (1990), Kawamura, Niiyama, and Niiyama (2009), Lassell, Fields, et al. (2022), Swall et al. (2015, 2016, 2019)	High confidence	No or very minor concerns regarding adequacy, and minor concerns regarding methodological limitations, coherence and relevance
Finding 11: Staff, volunteers and carers of people with dementia may observe very positive benefits of AAI for the person living with dementia; and reported a hope for the programmes to continue to see the long‐term impact	Casey et al. (2018), Fields (2017), Fields, Wood, and Lassell (2019), Hodgson et al. (2022), Kongable, Stolley, and Buckwalter (1990), Lassell, Cross, et al. (2022), Lassell, Fields, et al. (2022), Swall (2015), Swall et al. (2016, 2019)	High confidence	No or very minor concerns regarding coherence, and minor concerns regarding methodological limitations, relevance and adequacy
**Theme 3: Nurturing: Making AAI work**			
*Subtheme 1: Individualised and holistic approach*			
Finding 12: AAI needs to be a flexible and adaptive intervention providing various activities delivered tailored to individual needs	Busselman (2017), Casey et al. (2018), Fields (2017), Fields, Wood, and Lassell (2019), Hodgson et al. (2022), Kongable, Stolley, and Buckwalter (1990), Kawamura, Niiyama, and Niiyama (2009), Lassell, Cross, et al. (2022), Lassell, Fields, et al. (2022), Swall (2015), Swall et al. (2016)	High confidence	No or very minor concerns regarding coherence and adequacy, and minor concerns regarding methodological limitations and relevance
Finding 13: A multicomponent AAI programme can provide additional everyday activities and a rich holistic experience for people with dementia	Busselman (2017), Fields (2017), Fields, Wood, and Lassell (2019), Hodgson et al. (2022), Lassell, Cross, et al. (2022), Lassell, Fields, et al. (2022)	Moderate confidence	No or very minor concerns regarding coherence, minor concerns regarding methodological limitations and adequacy and moderated concerns regarding relevance
*Subtheme 2: Training and support*			
Finding 14: Experienced staff in AAI are valuable, conducting training around dementia care and AAI to improve staff's knowledge and skills is necessary	Fields (2017), Fields, Wood, and Lassell (2019), Kongable, Stolley, and Buckwalter (1990)	Moderate confidence	No or very concerns regarding coherence, and moderate concerns regarding methodological limitations, relevance and adequacy
Finding 15: There may be associated risks for people with dementia engaging in AAI, which may be reduced through safety measures or support from staff and care partners	Busselman (2017), Fields (2017), Fields, Wood, and Lassell (2019), Hodgson et al. (2022), Kongable, Stolley, and Buckwalter (1990), Lassell, Cross, et al. (2022), Swall (2015), Swall et al. (2016)	High confidence	No or very minor concerns regarding coherence, minor concerns regarding methodological limitations and adequacy and moderate concerns regarding relevance

### 3.8 Review Author Reflexivity

The research team engaged in prospective and retrospective reflexivity throughout all stages of the process. This involved considering how researcher subjectivity (briefs, background, value system and knowledge base) could influence each step of the research. The review team has a shared love of animals and positive attitude to the delivery of AAI for people with dementia.

The review team members' various backgrounds and expertise strengthened the reflexivity with different perspectives and practices, including general and children's nursing (CH), geriatric nursing (DZ), health promotion/health services research (MH) and rehabilitation therapy/health sciences (QL). All authors have clinical, academic and/or personal experience working with people with dementia, and have training in qualitative research. CH and MH have experience in qualitative evidence synthesis and thematic synthesis. CH is a co‐convenor of Cochrane Qualitative and Implementation Methods Group (QIMG). The review also incorporated Patient and Public Involvement (PPI); a man with dementia and his wife who have a dog named Teddy and share a love of dogs, advised each step of the review, providing insights and perspectives which also enhanced our reflexivity. Regular meetings, discussions and decisions were recorded. A reflexive journal was documented by the lead author (DZ) throughout the review process.

## 4 Results

We included 14 reports from 11 studies in our review (see Figure 1).

### 4.1 Description of the Studies

The included studies (Table 1) were published in English between 1990 and 2022. Studies included 148 participants, including people with dementia (*n* = 62), care partners/family members (terms used interchangeably but herein will be referred to as ‘care partners’) (*n* = 10), nursing staff or service providers involved in the AAI programme (*n* = 76). While studies were from a range of countries/geographical areas, including United States (*n* = 5), Sweden (*n* = 3) and United Kingdom, Canada and Japan individually. Study settings were spread over 25 residential care settings/nursing homes, and four therapeutic riding centres (visited by people with dementia who resided in nursing homes or local dementia and aging‐oriented organisations within the community).

The AAI programmes in the studies used a variety of animals, including dogs (*n* = 5), horses/ponies/donkeys (*n* = 4), hens (*n* = 1) and a mixed group of animals (sheep, rabbits, goat and chickens) (*n* = 1). All studies included activities involving interactions with animals through petting, grooming, touching and talking. Two studies had animal‐related creative activities such as painting, singing songs or telling stories about animals. Four studies encompassed equine‐assisted interventions, including activities such as horse riding.

### 4.2 Review Findings

The overarching theme of our review is ‘connection’ which describes the perceptions and experiences of AAI for people with dementia. We developed three analytical themes with an analogy of planting, growing and nurturing to describe the connection: (1) planting–connecting with animals, (2) growing–engaging in AAI and (3) nurturing–making AAI work (Table 4). From the themes/subthemes, we identified 15 key findings which are presented in Table 3, and supporting primary data can be found in File S5.

**TABLE 4:** Analytical and descriptive themes of the findings with analogy. [Colour table can be viewed at wileyonlinelibrary.com]

Gardening analogy	Analytical themes	Subthemes
**Planting** 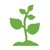	Connecting with animals	Willingness to connect
		Building relationships
**Growing** 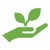	Engaging in AAI	A rich experience
		The benefits of AAI
**Nurturing** 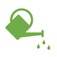	Making AAI work	Individualised and holistic approach
		Training and support

*Note:* Images were searched from the Icons in PowerPoint of Microsoft Office Home and Student 2019.

### 4.3 Theme 1: Planting: Connecting With Animals

This theme identifies the initial planting of the connections in AAI. It examines how people with dementia connect with animals and build relationships with animals and others. The subthemes are ‘willingness to connect’ and ‘building relationships’.

#### 4.3.1 Subtheme 1: Willingness to Connect

This subtheme describes people with dementia, their care partners and staff's willingness to connect with AAI and the animals; and how it may change over time.

The majority of people with dementia expressed their affection for, and interest in, animals, and previous experience with animals facilitated involvement (Fields, Wood, and Lassell 2019; Fields 2017; Hodgson et al. 2022; Kawamura, Niiyama, and Niiyama 2009; Kongable, Stolley, and Buckwalter 1990; Lassell, Fields, et al. 2022). Five staff in the RM (Riding in the Moment) programme reported that people with dementia were eager to engage with AAI because they believed interacting with horses is enticing (Fields, Wood, and Lassell 2019; Fields 2017). Conversely, low interest in animals diminishes participants' willingness to engage, as a dog handler shared that a man was forced to attend AAI by his ‘stubborn’ wife and he always rejected and cancelled animal visits (Swall et al. 2016). Therefore, recognising who might dislike animals when planning AAI is necessary (Kongable, Stolley, and Buckwalter 1990; Swall et al. 2016).

Most staff and care partners also expressed positive attitudes towards AAI (Casey et al. 2018; Fields, Wood, and Lassell 2019; Fields 2017; Hodgson et al. 2022; Kongable, Stolley, and Buckwalter 1990). The presence of animals aroused curiosity and interest throughout the nursing home. The expectation of care partners and staff of the potential positive impacts for people with dementia from engaging in AAI contributed to their willingness to be involved. Care partners also expressed their hope that AAI would enable their loved one with dementia to be independent of doing things (Lassell, Cross, et al. 2022). However, a few staff still stated negative attitudes towards AAI and said it was ‘a waste of time’; which was attributed to feelings of burnout and long working hours (Casey et al. 2018; Fields, Wood, and Lassell 2019; Fields 2017).

In some instances, despite initial resistance, the person with dementia's willingness towards AAI increased after engaging in a few activity sessions, and staff played an important role in encouraging people with dementia to try and connect with animals (Fields 2017; Hodgson et al. 2022). For example, a riding instructor shared that one woman ‘was not getting into any of the activities initially’, but she soon enjoyed the RM programme after trying (Fields 2017). Similarly, another woman in a care home who was reticent about touching hens, with staff's encouragement, she went from ‘I just want to look at them (hens)’ to actively ‘go and get the chicken’, and then placed a towel on her knees for the hen to sit on, as observed in the study (Hodgson et al. 2022).

#### 4.3.2 Subtheme 2: Building Relationships

This subtheme describes how relationships may develop from the planting of the original connections. These relationships can be developed between people with dementia and animals, and between people with dementia and other people.

Studies found that trained animals facilitated building a therapeutic relationship with people with dementia (Fields, Wood, and Lassell 2019; Fields 2017; Kongable, Stolley, and Buckwalter 1990; Lassell, Fields, et al. 2022; Swall et al. 2015, 2017). Horses can be attuned to people with dementia, and may have an underlying capacity for empathy, as a riding instructor stated, ‘I have a horse that may be naughty in some situations but once they (horse) get around the elderly…they are just calmer and that helps the residents relax.’ (Fields, Wood, and Lassell 2019; Fields 2017). A similar finding was observed in Swall et al. (2015, 2017), where the dog understood the person's limitations and responded positively to them. People with cognitive impairment may be ‘causing a stress overload for the animal’, through ‘obedience training’ the dog learned to understand cues of aggressive behaviour thus minimising any potential harm (Kongable, Stolley, and Buckwalter 1990).

People with dementia may also begin to feel a sense of protectiveness and responsibility for caring for the animals in AAI (Hodgson et al. 2022; Kawamura, Niiyama, and Niiyama 2009; Swall 2015; Swall et al. 2019, 2015, 2017). An observation of dog therapy showed that participants were being empathic and affectionate towards the dog; they treated the dog as ‘fragile’ and ‘vulnerable’ friend (Swall et al. 2015, 2017). One‐on‐one interaction can enable an emotional connection to an animal, facilitating special and unique relationships (Hodgson et al. 2022; Kawamura, Niiyama, and Niiyama 2009; Lassell, Cross, et al. 2022).

The presence of animals may provide an opportunity to enhance social relationships through mutual interests and shared experience of animals among people involved in AAI (Fields, Wood, and Lassell 2019; Fields 2017; Hodgson et al. 2022; Kawamura, Niiyama, and Niiyama 2009; Kongable, Stolley, and Buckwalter 1990; Lassell, Fields, et al. 2022; Swall et al. 2016). Dogs could facilitate a strengthened bond between people with dementia and volunteers, thus building trusting relationships (Kawamura, Niiyama, and Niiyama 2009). An inclusive space can be created with closer relationships among people with dementia, staff and care partners, and invoke a sense of belonging by sharing similar encounters or experiences in AAI (Fields, Wood, and Lassell 2019; Fields 2017; Kongable, Stolley, and Buckwalter 1990; Lassell, Fields, et al. 2022).

### 4.4 Theme 2: Growing: Engaging in AAI

This theme examines the continued growing of relationships, various interactions and the potential benefits of engaging in AAI. The subthemes are ‘a rich experience’ and ‘the benefits of AAI’.

#### 4.4.1 Subtheme 1: A Rich Experience

This subtheme describes the rich experiences within AAI and how they contributed to the engagement and growing relationships.

Most studies reported interactions between people with dementia and animals were multidimensional experiences; from distant sensory input to closer physical and cognitive stimulations (Busselman 2017; Fields, Wood, and Lassell 2019; Fields 2017; Hodgson et al. 2022; Kongable, Stolley, and Buckwalter 1990; Lassell, Cross, et al. 2022; Lassell, Fields, et al. 2022; Swall 2015; Swall et al. 2019, 2015, 2016, 2017). The sensory stimulation input in the RM programme was described as ‘smelling’ and ‘hearing’ horses (Fields, Wood, and Lassell 2019; Fields 2017). Some people with dementia engaged in AAI simply by watching (Hodgson et al. 2022; Kongable, Stolley, and Buckwalter 1990). Physical proximity and contact such as ‘grooming’, ‘petting’ and ‘cuddling’ were seen as nonverbal interactions with animals (Fields, Wood, and Lassell 2019; Fields 2017; Hodgson et al. 2022; Kongable, Stolley, and Buckwalter 1990; Lassell, Fields, et al. 2022; Swall 2015; Swall et al. 2019, 2017). Even for people with dementia at end‐of‐life, the ‘silent companionship’ of a dog could create a calm atmosphere and facilitate a peaceful death with reduced pain, symptoms and hyperventilation (Swall et al. 2019, 2016). Advanced activities in AAI can provide a higher‐level interaction for participants to maintain physical and cognitive functions (Busselman 2017; Fields, Wood, and Lassell 2019; Fields 2017; Kongable, Stolley, and Buckwalter 1990; Swall et al. 2019). Such as following the assistant's instruction to manage multiple tasks (Fields, Wood, and Lassell 2019; Fields 2017), or engaging in creative activities such as ‘designing…henhouses, singing about hens, painting, …and poetry…’ (Hodgson et al. 2022).

Interacting with animals could also evoke memories related to past experiences of own pets, childhood, significant places and important people (Fields, Wood, and Lassell 2019; Fields 2017; Kawamura, Niiyama, and Niiyama 2009; Kongable, Stolley, and Buckwalter 1990; Lassell, Fields, et al. 2022; Swall 2015; Swall et al. 2015, 2016, 2017). This could awaken temporary presence of mind and sense of self‐awareness (Swall 2015; Swall et al. 2015, 2017). Recalled memories could also reveal more about the identities of people with dementia, like ‘farms’ or ‘ranchers’; those memories ‘they couldn't remember until they were exposed to it again’ (Fields, Wood, and Lassell 2019; Fields 2017). Recent memories could also be triggered when participants looked at the pictures of AAI (Lassell, Fields, et al. 2022). Sometimes, difficult memories can be recalled by people with dementia and cause negative expressions (Kawamura, Niiyama, and Niiyama 2009; Swall 2015; Swall et al. 2015, 2016). In addition, temporary presence of mind challenged people with dementia to balance memory and reality (Kongable, Stolley, and Buckwalter 1990; Swall 2015; Swall et al. 2015). For example, Kongable, Stolley, and Buckwalter (1990) reported that ‘a few participants mistakenly called the therapy dog by the name of their personal pet’. This confusion could result in a sense of uncertainty and fear, bringing upset and sadness, but it could be minimised by the presence of therapy animals (Swall et al. 2015).

Furthermore, AAI provided opportunity for social interactions with other people such as staff and care partners (Casey et al. 2018; Fields, Wood, and Lassell 2019; Fields 2017; Hodgson et al. 2022; Kawamura, Niiyama, and Niiyama 2009; Kongable, Stolley, and Buckwalter 1990; Lassell, Cross, et al. 2022; Lassell, Fields, et al. 2022; Swall 2015; Swall et al. 2019, 2015, 2016, 2017). Animals created a nonthreatening atmosphere and conversation starter (Hodgson et al. 2022; Kawamura, Niiyama, and Niiyama 2009; Kongable, Stolley, and Buckwalter 1990), and could trigger deeper and serious topics around life and death, which helped people with dementia to ‘open up’ (Swall et al. 2019, 2016). AAI also provided the opportunity to connect with nature outdoors and indoors, which was beneficial for the well‐being of people with dementia and for environmental improvements of care facilities (Fields, Wood, and Lassell 2019; Fields 2017; Hodgson et al. 2022; Lassell, Cross, et al. 2022).

#### 4.4.2 Subtheme 2: The Benefits of AAI

This theme relates to the perceived psychological, physical, cognitive and social benefits of engaging in AAI.

Most people with dementia experienced great enjoyment and positive feelings during the AAI (Fields 2017; Hodgson et al. 2022; Kawamura, Niiyama, and Niiyama 2009; Kongable, Stolley, and Buckwalter 1990; Swall 2015; Swall et al. 2015, 2017). Staff and care partners also repeatedly reported that people with dementia experience positive emotions and moods (Casey et al. 2018; Fields, Wood, and Lassell 2019; Hodgson et al. 2022; Kongable, Stolley, and Buckwalter 1990; Lassell, Cross, et al. 2022; Lassell, Fields, et al. 2022), and may feel a sense of excitement, contentment, accomplishment and confidence when engaging in AAI.

Engaging in AAI may enable people with dementia to maintain or strengthen physical function, and reduce the symptoms of dementia (Fields, Wood, and Lassell 2019; Fields 2017; Hodgson et al. 2022; Kongable, Stolley, and Buckwalter 1990; Lassell, Fields, et al. 2022; Swall 2015; Swall et al. 2019, 2016, 2017). Physical improvements in body control, strength, balance and endurance were perceived by staff and care partners in RM programme (Fields, Wood, and Lassell 2019; Fields 2017; Lassell, Fields, et al. 2022). Animals gave participants motivation to be physically active. A similar unexpected experience of a dying person was observed in Swall et al. (2019), where he patted the dog using the arm he had not used for very long time, to the surprise of his physiotherapist and daughter. Additionally, AAI encouraged additional exercise; a daughter reported her mother began to participate in ‘water aerobics’ independently after AAI (Lassell, Fields, et al. 2022).

Improvement in short‐term memory, orientation, executive function, attention and sequencing capacities were noted in several studies as result of engaging in AAI (Casey et al. 2018; Fields, Wood, and Lassell 2019; Fields 2017; Hodgson et al. 2022; Kongable, Stolley, and Buckwalter 1990; Lassell, Fields, et al. 2022). AAI helped people with dementia to concentrate and focus on tasks, increase awareness of their surrounding environment and reduce agitation, depression, irritation, stress and wandering (Casey et al. 2018; Fields, Wood, and Lassell 2019; Fields 2017; Hodgson et al. 2022; Kawamura, Niiyama, and Niiyama 2009; Kongable, Stolley, and Buckwalter 1990; Swall et al. 2019). Improvements in speech function and social capacity were also reported, with people with dementia becoming more ‘clear’, ‘fluent’, ‘accurate’ and saying ‘complete sentences’ (Fields, Wood, and Lassell 2019; Fields 2017; Hodgson et al. 2022; Kawamura, Niiyama, and Niiyama 2009; Kongable, Stolley, and Buckwalter 1990; Lassell, Fields, et al. 2022; Swall et al. 2015, 2016, 2019).

Moreover, staff, volunteers and care partners perceived benefits beyond their expectations of AAI for people with dementia (Fields, Wood, and Lassell 2019; Fields 2017; Hodgson et al. 2022; Lassell, Cross, et al. 2022; Swall 2015; Swall et al. 2019, 2016). A daughter described ‘it is an amazing feeling’ of hearing that her nonverbal mother ‘had so many connections with people and animals in RM program’ (Fields, Wood, and Lassell 2019; Fields 2017). Positive comments related to the desire for the AAI to continue for more possible long‐term impacts (Casey et al. 2018; Hodgson et al. 2022; Kongable, Stolley, and Buckwalter 1990). Staff indicated that the impacts on people with dementia did not last long, mainly experienced the moment, and the author concluded more regular AAI would have further long‐term impacts (Casey et al. 2018). Swall et al. (2016) indicated that people's symptoms of dementia returned a few weeks after AAI visits. However, a care partner asserted that improvements to her mother's self‐esteem and confidence were sustained in her daily life after AAI (Lassell, Fields, et al. 2022).

### 4.5 Theme 3: Nurturing: Making AAI Work

Once connections are growing, it is important to examine how to nurture them. This theme examines the factors related to AAI that contribute to a successful complex intervention. The subthemes are ‘individualised and holistic approach’ and ‘training and support’.

#### 4.5.1 Subtheme 1: Individualised and Holistic Approach

This subtheme highlights that AAI needs to be an individualised and holistic intervention which can be delivered flexibly to meet individual needs and preferences.

A flexible and individualised AAI can provide various activities and be delivered through different approaches, tailoring to the needs, preferences and capacities of people with dementia (Busselman 2017; Fields, Wood, and Lassell 2019; Fields 2017; Hodgson et al. 2022; Swall 2015; Swall et al. 2016). Knowing and being familiar with the person with dementia and being able to provide bespoke AAI is essential for more engagement. As a director of the RM programme outlined, ‘everybody has their preferences and we pride ourselves in knowing our residents and allowing them to decide what they want’ (Fields, Wood, and Lassell 2019; Fields 2017). Likewise, dog handlers used their knowledge of people with dementia to make ‘ethical and liberal’ decisions on their AAI visits, which was helpful in reducing difficult situations (Swall 2015; Swall et al. 2016). It was important to consider the benefits of individual versus group interactions (Hodgson et al. 2022; Kawamura, Niiyama, and Niiyama 2009; Casey et al. 2018); and whether to include small animals that could be brought indoors versus horse riding which was weather dependent (Hodgson et al. 2022; Lassell, Cross, et al. 2022).

A multicomponent AAI programme offering additional everyday activity is the foundation of a flexible AAI and a holistic experience for people with dementia (Busselman 2017; Fields, Wood, and Lassell 2019; Fields 2017; Hodgson et al. 2022; Lassell, Cross, et al. 2022; Lassell, Fields, et al. 2022). The RM programme, for example, started with getting on the bus to riding centre, putting on and taking off helmets, human–horse interactions and additional social interactions like dancing and chatting (Busselman 2017; Fields, Wood, and Lassell 2019; Fields 2017; Lassell, Cross, et al. 2022; Lassell, Fields, et al. 2022). One instructor described it as ‘…having this kind of recipe ‐ those things together, I think are what makes the program so successful. I think if you took the elements apart individually you would not have that rich experience’ (Fields, Wood, and Lassell 2019; Fields 2017).

#### 4.5.2 Subtheme 2: Training and Support

This subtheme identifies the need for additional training and support to nurture or maintain the implementation of AAI.

Fields, Wood, and Lassell (2019) noted that there was a lack of formal volunteers and staff working with AAI and people with dementia, and only two staff in Kongable, Stolley, and Buckwalter (1990) had done some reading regarding AAI. Staff in the RM programme emphasised the need for more training and materials in relation to knowledge and skills of AAI, safety management and dementia care (Fields, Wood, and Lassell 2019; Fields 2017). Learning about AAI programmes before its commencement is important, as one staff member purposely visited the therapeutic riding centre and met with the director to learn how to operate the centre and access different people with dementia (Fields 2017).

Disability and behaviour changes associated with cognitive impairment could place people at higher risk when engaging in AAI (Busselman 2017; Fields, Wood, and Lassell 2019; Fields 2017). Kongable, Stolley, and Buckwalter (1990) identified five risks involved with AAI: hazard to the dog, hazard to participant, sensory overload, dislike of the animal and responsibility placed on nursing staff. Training for animals is necessary and can minimise potential risks (Kongable, Stolley, and Buckwalter 1990; Swall 2015; Swall et al. 2015, 2016, 2017). Identified risks for people with dementia were falling, being bitten, possible transmission of disease and overload sensory from animals (Kongable, Stolley, and Buckwalter 1990). Developing and implementing safety measures is helpful to ensure a safe AAI experience, for example, an emergency plan, wearing protective gear, understanding and supporting participants' needs, noticing environmental factors and providing staff/volunteer supervision (Fields, Wood, and Lassell 2019; Fields 2017; Hodgson et al. 2022). Care partner involvement was valuable in creating a comfortable atmosphere and providing emotional support for people with dementia (Lassell, Cross, et al. 2022; Lassell, Fields, et al. 2022). As one care partner reported ‘it made the experience much more enjoyable not just for me but for her to know that I'm right there the whole time, and talking, and being with’ (Lassell, Cross, et al. 2022).

## 5 Discussion

This review identified a core theme of ‘connection’ to examine key stakeholders' perceptions and experiences of AAI for people with dementia. Through the analytical themes, we developed a gardening analogy describing the process of planting, growing and nurturing these connections. We identified 15 key findings, which reflect the growth of connection through AAI, highlighting benefits, potential challenges and considerations for enhancing experiences and impacts of AAI.

Our synthesis found that most people with dementia, care partners and service providers were positively receptive towards AAI based on their prior experiences with animals. As suggested by Yakimicki et al. (2019), it may be necessary to assess individuals prior to introducing them to AAI as they may dislike or have an allergy to animals. We also found that while initially some people with dementia had low interest in AAI, they engaged nonetheless with subsequent enjoyment (Fields 2017; Hodgson et al. 2022). Cohen‐Mansfield, Dakheel‐Ali, and Marx (2009); Cohen‐Mansfield, Hai, and Comishen (2017) developed two models (the comprehensive process model of (group) engagement) to examine factors influencing the engagement of individual or group activities for people with dementia, including environmental attributes, personal attributes and stimuli attributes. Participants' interests, cognitive function and medication may affect their level of engagement, which can influence the impacts of AAI (Badin, Bailly, and Pennequin 2024; Olsen et al. 2016).

According to our findings, engaging in AAI is a multidimensional sensory experience, stimulating various physical and cognitive interactions which link to stimuli attributes of affecting engagement identified by Cohen‐Mansfield, Dakheel‐Ali, and Marx (2009); Cohen‐Mansfield, Hai, and Comishen (2017) in their models. Our review noted the importance of animals' presence as a stimulus, providing tactile, auditory, olfactory, visual and cognitive stimulation which can trigger memories and facilitate physical activities and social interactions. Previous studies outlined that people with dementia are more likely to experience sensory deprivation which challenges their health and well‐being. Sensory enhancement can compensate for unmet needs, thus reducing behaviours of concern and improving quality of life (Cohen‐Mansfield 2001; Jakob and Collier 2017). Providing meaningful activities to people with dementia through physical and social stimulation is an essential mechanism for a successful AAI (Shoesmith, Surr, and Ratschen 2023).

Most studies in our review reported very positive experiences and impacts of AAI for people with all stages of dementia, prompting improvement of psychological, physical, social and cognitive function, but our findings could not draw comparisons across different stages of dementia within the existing data. Shoesmith, Surr, and Ratschen (2023) identified group activities as suitable for people with mild dementia, but individual activity may be necessary for people with advanced dementia, as they may have limited ability for group interactions (Olsen, Pedersen, Bergland, Enders‐Slegers, and Ihlebæk 2016). Nevertheless, improvement in social behaviour is not limited by the stage of dementia (Quintavalla et al. 2021). Moreover, it could be argued that AAI may improve cognitive function of people with dementia, including memory, orientation, executive function and attention, and reduce behaviours of concern as described in the studies by Casey et al. (2018), Fields (2017) and Hodgson et al. (2022). This is in contrast to the systematic reviews (Batubara et al. 2022; Chen et al. 2022; Zafra‐Tanaka et al. 2019), which did not observe effects of AAI on cognitive function, possibly due to the variations in sample size and methodologies between qualitative and quantitative studies (Orr et al. 2023).

Pets are often regarded as family members or friends; the presence of personal pets and therapy animals facilitates emotional human–animal bonds (Barchas et al. 2020; Shoesmith, Surr, and Ratschen 2023). Like other studies (MacNamara, Moga, and Pachel 2019; Fine 2019), our review found that therapy animals can diminish potential risks, promote safe interactions and foster human–animal relationships, which contribute to achieving therapeutic goals (Kongable, Stolley, and Buckwalter 1990; Swall et al. 2017). Interaction with certain animals can facilitate a comfortable relationship, and people with dementia may feel a sense of protectiveness towards the animals (Kawamura, Niiyama, and Niiyama 2009; Swall 2015), which motivates engagement in AAI (Peluso et al. 2018; Ritchie et al. 2019). We found that small‐size animals are more flexible to space and location (Swall 2015), residential animals tend to provide sustained stimulation and long‐term impacts (Hodgson et al. 2022), and activities involving large animals, conducted outdoors, are dependent on space and weather conditions (Fields 2017). Machová et al. (2020) noted that small‐ or medium‐size dogs seems preferable to larger ones. Regardless of species and breed, animal type can depend on recipients' preferences and therapeutic needs (MacNamara, Moga, and Pachel 2019).

The findings of our review highlight the importance of a flexible AAI, tailored to people with dementia's capacities, experiences and needs, as also illustrated by Olsen, Pedersen, Bergland, Enders‐Slegers, Patil, et al. (2016). This can be realised through diverse approaches and individualised animal visit schedules, providing additional everyday activities beyond daily routines and empowering participants to make decisions. Adequate screening of animals, and being familiar with people with dementia in advance of implementation can help to ensure safe and enjoyable AAI. Moreover, we identified a lack of training regarding AAI and dementia care for staff/volunteers who facilitate AAI, as similarly identified by Badin, Bailly, and Pennequin (2024). Risks of injury and unhealthy interactions may raise without appropriate training for animals and practitioners (e.g., training in animal health and behaviour, reading animal body language and infection control practices) (Fine 2019). In addition, involving veterinarians and animal handlers in AAI to ensure safety and well‐being of both people and animals is important. For our nonhuman therapy partner, practitioners should be aware of the ethical considerations, welfare and humane treatment of involved animals (Fine 2019).

Another concern was raised by Freeman and Linder (2019) regarding the lack of relevant policies or procedures for bringing animals into nursing facilities, and without human or animal health regulatory agencies for monitoring AAI programmes. Nurses can be considered as gatekeepers for incorporating AAI in dementia care and daily routine, through directly or cooperatively working in human–animal teams to introduce, implement and regulate AAI within facilities (Buettner, Fitzsimmons, and Barba 2011). Nursing leaders are largely proficient in making interprofessional decisions, and are encouraged to determine the strategic organisational planning, responsibility, workload and shift regarding AAI in line with local regulatory requirements (Abate 2020; Fine 2019). When nurses adopt AAI in community care settings, the issues mentioned above should be considered.

### 5.1 Limitations

Our review rigorously and systematically explored the experiences and perceptions of AAI with moderate to high confidence findings, but with some limitations. We included a moderate range of geographic spread, mostly from the United States and Sweden, which limits the transferability of our findings to other settings globally. The review included various types of animals in AAI, and it was difficult to make direct comparisons across different types of animals. Shoesmith, Surr, and Ratschen (2023) also reported the difficulty of comparing effectiveness of AAI by animal type, and difficulty of developing guidelines for best practices of AAI due to variations in intervention components. Our review incorporated the perspectives of a range of stakeholders, but we had limited data from people with dementia. Further research to better understand the views of people with dementia is needed (Phillipson and Hammond 2018).

## 6 Conclusion

AAI has potential benefits for people with dementia, and it is important to consider the qualitative evidence of implementing AAI, to facilitate engagement and long‐term impacts. We highlight planting, growing and nurturing connections among people with dementia, animals and other people. People's prior experiences and interests towards animals may affect building connections with therapy animals initially; a structured, multicomponent and individualised AAI, providing various additional everyday activities with sensory input, physical and social interactions and cognitive stimulation, is important to enhance engagement during AAI; additional training and materials for staff around AAI and dementia care are necessary pre‐implementation activities. Research into AAI for people with dementia needs to be conducted in other cultural, geographical and income‐level settings. Future research with longitudinal designs, and qualitative research with people with dementia using innovative approaches, is needed to better understand how to best deliver and realise the impacts of AAI for people with dementia.

## Author Contributions

**Dou Zhang:** conceptualisation, methodology, software, data curation, formal analysis, writing – review and editing. **Marita Hennessy:** conceptualisation, methodology, software, resources, writing – review and editing, supervision. **Qiuxia Li:** data curation, writing – review and editing. **Nuala Paley:** formal analysis, writing – review and editing. **Gerry Paley:** formal analysis, writing – review and editing. **Catherine Houghton:** conceptualisation, methodology, software, formal analysis, resources, writing – review and editing, supervision, funding acquisition.

## Ethics Statement

The authors have nothing to report.

## Consent

The authors have nothing to report.

## Conflicts of Interest

The authors declare no conflicts of interest.

## Supporting information


Appendix S1

## Data Availability

The data that supports the findings of this study are available in the Supporting Information of this article.
