# Supplementary material for: Perceptions and Experiences of Animal‐Assisted Interventions for People Living With Dementia: A Qualitative Evidence Synthesis
Source: J Clin Nurs. 2024 Oct 6;35(5):2517–36. doi: 10.1111/jocn.17429 (PMC13068170; doi:10.1111/jocn.17429)
Supplement: Supplementary file 1 — Appendix S1 [file JOCN-35-2517-s001.docx]

**Supplementary File 1. Enhancing Transparency in Reporting the Synthesis of Qualitative Research: ENTREQ checklist**

| **No** | **Item** | **Guide and Description** | **Included in Manuscript** |
| --- | --- | --- | --- |
| 1 | Aim | State the research question the synthesis addresses. | P. 2-3 & Supplementary File 2 |
| 2 | Synthesis methodology | Identify the synthesis methodology or theoretical framework which underpins the synthesis, and describe the rationale for choice of methodology *(e.g. meta-ethnography, thematic synthesis, critical interpretive synthesis, grounded theory synthesis, realist synthesis, meta-aggregation, meta-study, framework synthesis).* | P. 5 |
| 3 | Approach to searching | Indicate whether the search was pre-planned (*comprehensive search strategies to seek all available studies)* or iterative (*to seek all available concepts until they theoretical saturation is achieved)*. | P. 4 |
| 4 | Inclusion criteria | Specify the inclusion/exclusion criteria *(e.g. in terms of population, language, year limits, type of publication, study type).* | P. 3 |
| 5 | Data sources | Describe the information sources used (e.g. *electronic databases (MEDLINE, EMBASE, CINAHL, psycINFO, Econlit), grey literature databases (digital thesis, policy reports), relevant organisational websites, experts, information specialists, generic web searches (Google Scholar) hand searching, reference lists)* and when the searches conducted; provide the rationale for using the data sources. | P. 4 |
| 6 | Electronic Search strategy | Describe the literature search *(e.g. provide electronic search strategies with population terms, clinical or health topic terms, experiential or social phenomena related terms, filters for qualitative research, and search limits)*. | P. 4 & Supplementary File 3 |
| 7 | Study screening methods | Describe the process of study screening and sifting *(e.g. title, abstract and full text review, number of independent reviewers who screened studies).* | P. 4 |
| 8 | Study characteristics | Present the characteristics of the included studies *(e.g. year of publication, country, population, number of participants, data collection, methodology, analysis, research questions).* | P. 6 & Table 1 |
| 9 | Study selection results | Identify the number of studies screened and provide reasons for study exclusion *(e,g, for comprehensive searching, provide numbers of studies screened and reasons for exclusion indicated in a figure/flowchart; for iterative searching describe reasons for study exclusion and inclusion based on modifications t the research question and/or contribution to theory development).* | P.6 & Figure 1 |
| 10 | Rationale for appraisal | Describe the rationale and approach used to appraise the included studies or selected findings *(e.g. assessment of conduct (validity and robustness), assessment of reporting (transparency), assessment of content and utility of the findings).* | P. 4-5 |
| 11 | Appraisal items | State the tools, frameworks and criteria used to appraise the studies or selected findings *(e.g. Existing tools: CASP, QARI, COREQ, Mays and Pope* [[25](https://link.springer.com/article/10.1186/1471-2288-12-181#ref-CR25)]*; reviewer developed tools; describe the domains assessed: research team, study design, data analysis and interpretations, reporting).* | P. 4-5, CASP |
| 12 | Appraisal process | Indicate whether the appraisal was conducted independently by more than one reviewer and if consensus was required. | P. 4-5 |
| 13 | Appraisal results | Present results of the quality assessment and indicate which articles, if any, were weighted/excluded based on the assessment and give the rationale. | P. 5 & Table 2 |
| 14 | Data extraction | Indicate which sections of the primary studies were analysed and how were the data extracted from the primary studies? *(e.g. all text under the headings “results /conclusions” were extracted electronically and entered into a computer software).* | P. 4 |
| 15 | Software | State the computer software used, if any. | P. 4-5, Covidence, Endnote, and NVivo |
| 16 | Number of reviewers | Identify who was involved in coding and analysis. | P. 5 |
| 17 | Coding | Describe the process for coding of data *(e.g. line by line coding to search for concepts).* | P. 5 |
| 18 | Study comparison | Describe how were comparisons made within and across studies *(e.g. subsequent studies were coded into pre-existing concepts, and new concepts were created when deemed necessary).* | Not applicable |
| 19 | Derivation of themes | Explain whether the process of deriving the themes or constructs was inductive or deductive. | P. 5 |
| 20 | Quotations | Provide quotations from the primary studies to illustrate themes/constructs, and identify whether the quotations were participant quotations of the author’s interpretation. | P. 7-12 & Supplementary File 5 |
| 21 | Synthesis output | Present rich, compelling and useful results that go beyond a summary of the primary studies (e.g. *new interpretation, models of evidence, conceptual models, analytical framework, development of a new theory or construct).* | p. 7-15, Table 3 & 4, Supplementary File 4 |

**Supplementary File 2. Review Question Formulation**

| **Review question: What are the experiences and perceptions of key stakeholders perceived to animal assisted intervention (AAI) for people with dementia in community care settings?**  (What are factors influencing the delivery of AAI for people with dementia? If there are any differences of AAI amongst different types of animals, settings, and stages of dementia?) | | | | |
| --- | --- | --- | --- | --- |
| **Setting** | **Perspective** | **Interest, phenomenon of** | **Comparison (optional)** | **Evaluation** |
| Community care settings (e.g., nursing homes, care homes, day care centres, therapy centre) | Perspective of stakeholders (e.g., people with dementia and their care partners, health care professionals, animal handlers) | Animal assisted intervention for people with dementia | Differences amongst types of animals, settings, and stages of dementia | Experiences, perceptions, attitudes, factors (facilitators and barriers) |

**Supplementary File 3. Search Strategy.**

**Medline (Ovid, searched 20/02/2022)**

| # | Searches | Results |
| --- | --- | --- |
| 1 | exp Animal Assisted Therapy/ | 804 |
| 2 | exp Equine-Assisted Therapy/ | 267 |
| 3 | exp Therapy Animals/ | 26 |
| 4 | exp Pets/ | 3232 |
| 5 | exp Human-Animal Bond/ | 1995 |
| 6 | exp Human-Animal Interaction/ | 2053 |
| 7 | (((animal$ or canine or pet$ or dog$ or horse$) and (assist* or therap* or facilitated)) or animal-assisted or ((companion or visit*) and animal$) or service animal program*).mp. | 1450830 |
| 8 | 1 or 2 or 3 or 4 or 5 or 6 or 7 | 1453980 |
| 9 | exp Dementia/ | 200206 |
| 10 | exp Alzheimer Disease/ | 115409 |
| 11 | exp Cognition Disorders/ | 113802 |
| 12 | exp Cognitive Dysfunction/ | 34131 |
| 13 | exp Neurocognitive Disorders/ | 304424 |
| 14 | (dement* or Alzheimer* or (Cognitive* adj3 (impair* or dysfunction*)) or cognitive decline).mp. | 376541 |
| 15 | 9 or 10 or 11 or 12 or 13 or 14 | 454486 |
| 16 | 8 and 15 | 43687 |
| 17 | limit 16 to "qualitative (best balance of sensitivity and specificity)" | 779 |

**CINAHL (EBSCOhost, searched 20/02/2023)**

| # | Searches | Results |
| --- | --- | --- |
| S1 | (MH "Pet Therapy+") | 1873 |
| S2 | (MH "Therapy Animals") | 31 |
| S3 | (MH "Equine-Assisted Therapy") | 345 |
| S4 | (MH "Service Animals") | 720 |
| S5 | (MH "Animal Assisted Therapy (Iowa NIC)" | 1 |
| S6 | (MH "Pets") | 3021 |
| S7 | (MH "Human-Pet Bonding") | 1013 |
| S8 | (MH "Human-Animal Interaction") | 55 |
| S9 | TI (((animal* or canine or pet? or dog? or horse*) and (assist* or therap* or facilitated)) or animal-assisted or ((companion or visit*) and animal?) or service animal program*) OR AB (((animal* or canine or pet? or dog? or horse*) and (assist* or therap* or facilitated)) or animal-assisted or ((companion or visit*) and animal?) or service animal program*) | 24793 |
| S10 | S1 OR S2 OR S3 OR S4 OR S5 OR S6 OR S7 OR S8 OR S9 | 29192 |
| S11 | (MH "Dementia+") | 84665 |
| S12 | (MH "Dementia, Senile+") | 39653 |
| S13 | (MH "Dementia, Presenile+") | 39314 |
| S14 | (MH "Dementia Patients") | 3692 |
| S15 | (MH "Alzheimer's Disease") | 37761 |
| S16 | (MH "Cognition Disorders+") | 36698 |
| S17 | TI ((dement* or Alzheimer* or Cognitive* N3 (impair* or dysfunction*) or cognitive decline) OR AB ((dement* or Alzheimer* or Cognitive* N3 (impair* or dysfunction*) or cognitive decline) | 123300 |
| S18 | S11 OR S12 OR S13 OR S14 OR S15 OR S16 OR S17 | 156953 |
| S19 | 10 AND 18 | 1273 |
| S20 | S19 Limiters - Clinical Queries: Qualitative - High Sensitivity | 234 |

**Embase (Ovid, searched 20/02/2023)**

| # | Searches | Results |
| --- | --- | --- |
| #1 | 'animal assisted therapy'/exp | 1579 |
| #2 | 'hippotherapy'/exp | 589 |
| #3 | 'therapy animal'/exp | 153 |
| #4 | 'pet animal'/exp | 8188 |
| #5 | 'human-animal bond'/exp | 882 |
| #6 | 'human-animal relation'/exp | 1183 |
| #7 | (animal$:ti,ab,kw OR canine:ti,ab,kw OR pet$:ti,ab,kw OR dog$:ti,ab,kw OR horse$:ti,ab,kw) AND (assist*:ti,ab,kw OR therap*:ti,ab,kw OR facilitated:ti,ab,kw) OR 'animal assisted':ti,ab,kw OR ((companion:ti,ab,kw OR visit*:ti,ab,kw) AND animal$:ti,ab,kw) OR 'service animal program*':ti,ab,kw | 340972 |
| #8 | #1 OR #2 OR #3 OR #4 OR #5 OR #6 OR #7 | 348261 |
| #9 | 'dementia'/exp | 429475 |
| #10 | 'Alzheimer disease'/exp | 243730 |
| #11 | dement*:ti,ab,kw OR alzheimer*:ti,ab,kw OR ('cognitive* adj3':ti,ab,kw AND (impair*:ti,ab,kw OR dysfunction*:ti,ab,kw OR decline:ti,ab,kw)) | 376963 |
| #12 | #9 OR #10 OR #11 | 504526 |
| #13 | 'qualitative research'/exp | 123660 |
| #14 | qualitative:ti,ab,kw OR ethnograph$:ti,ab,kw OR phenomenolog$:ti,ab,kw OR 'grounded theory':ti,ab,kw OR hermeneutic$:ti,ab,kw OR “experience$”:ti,ab,kw OR narrat$:ti,ab,kw OR 'action research':ti,ab,kw OR observation$:ti,ab,kw OR “focus group$”:ti,ab,kw OR interview$:ti,ab,kw OR 'mixed method':ti,ab,kw OR multimethod:ti,ab,kw OR descript*:ti,ab,kw | 3225302 |
| #15 | #13 OR #14 | 3233855 |
| #16 | #8 AND #12 AND #15 | 899 |

**APA PsycINFO (EBSCOhost, searched 20/02/2023)**

| # | Searches | Results |
| --- | --- | --- |
| S1 | DE "Animal Assisted Therapy" | 1347 |
| S2 | DE "Pets" | 2077 |
| S3 | DE "Service Animals" | 129 |
| S4 | TI (((animal* or canine or pet? or dog? or horse*) and (assist* or therap* or facilitated)) or animal-assisted or ((companion or visit*) and animal?) or service animal program*) OR AB (((animal* or canine or pet? or dog? or horse*) and (assist* or therap* or facilitated)) or animal-assisted or ((companion or visit*) and animal?) or service animal program*) | 19041 |
| S5 | S1 OR S2 OR S3 OR S4 | 20398 |
| S6 | **DE "Dementia"** OR DE "AIDS Dementia Complex" OR DE "Dementia with Lewy Bodies" OR DE "Presenile Dementia" OR DE "Pseudodementia" OR DE "Semantic Dementia" OR DE "Senile Dementia" OR DE "Vascular Dementia" | 53367 |
| S7 | **DE "Alzheimer's Disease"** | 54209 |
| S8 | (**DE "Cognitive Impairment"**) OR (DE "Neurocognitive Disorders" OR DE "Consciousness Disorders" OR DE "Delirium" OR DE "Dementia" OR DE "Memory Disorders" OR DE "Mild Cognitive Impairment") | 103596 |
| S9 | TI (dement* or Alzheimer* or Cognitive* N3 (impair* or dysfunction*) or cognitive decline) OR AB (dement* or Alzheimer* or Cognitive* N3 (impair* or dysfunction*) or cognitive decline) | 158292 |
| S10 | S6 OR S7 OR S8 OR S9 | 189049 |
| S11 | S5 AND S10 | 2784 |
| S12 | S17 Limiters - Methodology: QUALITATIVE STUDY | 26 |

**AgeLine (EBSCOhost, searched 20/02/2023)**

| # | Searches | Results |
| --- | --- | --- |
| S1 | DE "Pet Therapy" | 129 |
| S2 | DE "Pets" | 279 |
| S3 | TI ((animal$ or canine or pet$ or dog$ or horse$) and (assist* or therap* or facilitated) or animal-assisted or (companion or visit*) and animal$ or service animal program*) OR AB ((animal$ or canine or pet$ or dog$ or horse$) and (assist* or therap* or facilitated) or animal-assisted or (companion or visit*) and animal$ or service animal program*) | 478 |
| S4 | S1 OR S2 OR S3 | 666 |
| S5 | **DE "Dementia"** OR DE "Alzheimers Disease" OR DE "Early Onset Dementia" OR DE "Frontotemporal Dementia" OR DE "Lewy Body Dementia" OR DE "Vascular Dementia" | 24324 |
| S6 | DE "Alzheimers Disease" | 10855 |
| S7 | **DE "Cognitive Impairment"** OR DE "Memory Impairment" | 8748 |
| S8 | TI (dement* or Alzheimer* or (Cognitive* N3 (impair* or dysfunction*)) or cognitive decline) OR AB (dement* or Alzheimer* or (Cognitive* N3 (impair* or dysfunction*)) or cognitive decline) | 34655 |
| S9 | S5 OR S6 OR S7 OR S8 | 37022 |
| S10 | **DE "Qualitative Research"** OR DE "Case Studies" OR DE "Ethnography" OR DE "Focus Groups" OR DE "Life History Research" OR DE "Participant Observation" OR DE "Research Diaries" | 8808 |
| S11 | DE "Perception" | 1716 |
| S12 | DE "Narratives" | 555 |
| S13 | TI (qualitative OR ethnograph$ OR phenomenolog$ OR “grounded theory” OR hermeneutic$ OR “experience$” OR narrat$ OR “action research” OR observation$ OR “focus group$” OR interview$ OR “mixed method” OR multimethod OR descript*) OR AB (qualitative OR ethnograph$ OR phenomenolog$ OR “grounded theory” OR hermeneutic$ OR “experience$” OR narrat$ OR “action research” OR observation$ OR “focus group$” OR interview$ OR “mixed method” OR multimethod OR descript*) | 41790 |
| S14 | S10 OR S11 OR S12 OR S13 | 45472 |
| S15 | S4 AND S9 AND S14 | 40 |

**Web of Science (core collection, searched 20/02/2023)**

| # | Searches | Results |
| --- | --- | --- |
| 1 | TS=("animal assisted intervention*" OR "animal assisted therapy" OR "equine assisted therapy" OR "therapy animal*" OR pet* OR "human animal bond" OR "human animal interaction*" OR "pet* facilitate*”) | 662606 |
| 2 | AB=(((animal$ OR canine OR pet$ OR dog$ OR horse$) AND (assist* or therap* or facilitated)) OR animal-assisted OR ((companion or visit*) and animal$) OR service animal program*) | 229638 |
| 3 | #1 OR #2 | 862510 |
| 4 | TS=(dementia OR "Alzheimer Disease*" OR "Cognition Disorders" OR "Cognitive Dysfunction" OR "Cognitive impairment" OR “Neurocognitive Disorders” OR “cognitive decline”) | 315519 |
| 5 | AB=(dement* OR Alzheimer* OR (Cognitive* NEAR/3 (impair* OR dysfunction* OR decline))) | 303682 |
| 6 | #4 OR #5 | 407605 |
| 7 | TS=(qualitative OR ethnograph$ OR phenomenolog$ OR “grounded theory” OR hermeneutic$ OR “experience$” OR narrat$ OR “action research” OR observation$ OR “focus group$” OR interview$ OR “mixed method” OR multimethod OR descript*) | 5650906 |
| 8 | 3 AND 6 AND 7 | 1290 |

**Scopus (Elsevier, searched 20/02/2023)**

| # | Searches | Results |
| --- | --- | --- |
| 1 | TITLE-ABS-KEY ( ( ( animal$ OR canine OR pet$ OR dog$ OR horse$ ) AND ( assist* OR therap* OR facilitated ) ) OR animal-assisted OR ( ( companion OR visit* OR therapy) AND animal$ ) OR "service animal program*" OR "human animal bond" OR "human animal interaction*" OR pet* ) | 2190689 |
| 2 | TITLE-ABS-KEY ( dement* OR alzheimer* OR ( cognitive* W/3 ( impair* OR dysfunction* OR decline ) ) ) | 510114 |
| 3 | TITLE-ABS-KEY ( communit* OR residence* OR residential OR facilit* OR ( nursing W/3 ( home$ OR center* OR centre* ) ) OR ( day W/3 ( center* OR centre* OR care ) ) OR unit* OR "retirement home" OR institution* OR "Non hospital" OR "long term care" ) | 9921844 |
| 4 | TITLE-ABS-KEY ( qualitative OR ethnograph$ OR phenomenolog$ OR "grounded theory" OR hermeneutic$ OR "experience$" OR narrat$ OR "action research" OR observation$ OR "focus group$" OR interview$ OR "mixed method" OR multimethod OR descript* ) | 7247749 |
| 5 | 1 AND 2 AND 3 AND 4 | 380 |

**ProQuest (other resources, searched 20/02/2023)**

| # | Searches | Results |
| --- | --- | --- |
| S1 | MAINSUBJECT.EXACT("Animal assisted therapy") OR title((animal$ OR canine OR pet$ OR dog$ OR horse$) AND (assist* OR therap* OR facilitated)) OR title(animal-assisted OR “service animal program*” OR "Human Animal Bond" OR "Human Animal Interaction") OR title((companion OR visit* OR therapy) AND animal$) OR abstract((animal$ OR canine OR pet$ OR dog$ OR horse$) AND (assist* OR therap* OR facilitated)) OR abstract(animal-assisted OR “service animal program*” OR "Human Animal Bond" OR "Human Animal Interaction") OR abstract((companion OR visit* OR therapy) AND animal$) | 60682 |
| S2 | (MAINSUBJECT.EXACT("Dementia") OR MAINSUBJECT.EXACT("Neurological disorders") OR MAINSUBJECT.EXACT("Alzheimers disease")) OR title(dement* or Alzheimer*) OR abstract(dement* or Alzheimer*) OR title(Cognitive* N3 (impair* or dysfunction* or decline)) OR abstract(Cognitive* N3 (impair* or dysfunction* or decline)) | 259499 |
| S4 | (MAINSUBJECT.EXACT("Focus groups") OR MAINSUBJECT.EXACT("Qualitative research")) OR title(qualitative OR ethnograph$ OR phenomenolog$ OR “grounded theory” OR hermeneutic$ OR “experience$” OR narrat$ OR “action research” OR observation$ OR “focus group$” OR interview$ OR “mixed method” OR multimethod OR descript*) OR abstract(qualitative OR ethnograph$ OR phenomenolog$ OR “grounded theory” OR hermeneutic$ OR “experience$” OR narrat$ OR “action research” OR observation$ OR “focus group$” OR interview$ OR “mixed method” OR multimethod OR descript*) | 4354321 |
| S5 | S1 AND S2 AND S3 | 203 |

**PsycEXTRA (other resources, searched 20/02/2023)**

| # | Searches | Results |
| --- | --- | --- |
| S1 | DE "Animal Assisted Therapy" | 78 |
| S2 | DE "Pets" | 101 |
| S3 | DE "Service Animals" | 2 |
| S4 | TI ( (animal* or canine or pet? or dog? or horse*) and (assist* or therap* or facilitated) ) OR TI ( "animal-assisted" or "service animal program*" ) OR TI ( (companion or visit*) and animal? ) OR AB ( (animal* or canine or pet? or dog? or horse*) and (assist* or therap* or facilitated) ) OR AB ( "animal-assisted" or "service animal program*" ) OR AB ( (companion or visit*) and animal? ) | 379 |
| S5 | S1 OR S2 OR S3 OR S4 | 469 |
| S6 | DE "Dementia" OR DE "AIDS Dementia Complex" OR DE "Dementia with Lewy Bodies" OR DE "Presenile Dementia" OR DE "Pseudodementia" OR DE "Semantic Dementia" OR DE "Senile Dementia" OR DE "Vascular Dementia" | 719 |
| S7 | DE "Cognitive Impairment" | 926 |
| S8 | DE "Alzheimer's Disease" | 1257 |
| S9 | TI ( dement* or Alzheimer* ) OR TI ( Cognitive* N3 (impair* or dysfunction* or decline) ) OR AB ( dement* or Alzheimer* ) OR AB ( Cognitive* N3 (impair* or dysfunction* or decline) ) | 2849 |
| S10 | S6 OR S7 OR S8 OR S9 | 3327 |
| S11 | DE "Qualitative Methods" OR DE "Focus Group" OR DE "Grounded Theory" OR DE "Interpretative Phenomenological Analysis" OR DE "Narrative Analysis" OR DE "Semi-Structured Interview" OR DE "Thematic Analysis" | 261 |
| S12 | TI ( qualitative OR ethnograph$ OR phenomenolog$ OR “grounded theory” OR hermeneutic$ OR “experience$” OR narrat$ OR “action research” OR observation$ OR “focus group$” OR interview$ OR “mixed method” OR multimethod OR descript* ) OR AB ( qualitative OR ethnograph$ OR phenomenolog$ OR “grounded theory” OR hermeneutic$ OR “experience$” OR narrat$ OR “action research” OR observation$ OR “focus group$” OR interview$ OR “mixed method” OR multimethod OR descript* ) | 38629 |
| S13 | S5 AND S10 AND S11 | 2 |

**Search results**: 3853 records retrieved in total. 2895 left after deduplicating in Endnote. 2864 left after deduplicating in Covidence.

**Supplementary File 4. GRADE CERQual Full Evidence Profile**

| **Summary of review finding** | **Reports of studies contributing to the review finding** | **Methodological limitations of included studies** | **Coherence** | **Relevance** | **Adequacy** | **CERQual assessment of confidence in the evidence** | **Explanation of CERQual assessment** |
| --- | --- | --- | --- | --- | --- | --- | --- |
| **Theme 1: Planting: Connecting with animals** | | | | | | | |
| ***Subtheme 1: Willingness to connect*** | | | | | | | |
| Finding 1: The willingness of people with dementia, care partners and staff to engage with AAI may depend on prior experience or their attitude towards animals in general | Casey et al., 2018; Fields, 2017; Fields et al., 2019; Hodgson et al., 2022; Kawamura et al., 2009; Kongable et al., 1990; Lassell et al., 2022a; Lassell et al., 2022b; Swall et al., 2016 | **Minor concerns** based on the assessments of 1 study (1 reports) with no or very minor, 5 (6 reports) with minor, 1 with moderate, 1 with serious methodological limitations | **No or very minor concerns** | **Minor concerns**  regarding as studies had moderate geographical spread. Studies settings were conducted in both nursing homes and riding therapy centre  Participants had a range of stakeholders. People living with dementia had moderate range of diagnosis, age, and gender  Studies had a good time range | **No or very minor concerns** regarding moderately rich data | **High confidence** | No or very minor concerns regarding coherence and adequacy, and minor concerns regarding methodological limitations and relevance |
| Finding 2: Despite initial resistance, people with dementia may begin to engage with AAI after a few activities | Fields, 2017; Hodgson et al., 2022 | **Minor concerns** based on the assessments of 2 studies (2 reports) with minor methodological limitations | **No or very minor concerns** | **Moderate concerns**  regarding studies had limited geographical spread  Intervention had limited AAI activities and animals  Participants included people living with dementia and service providers | **Moderate concerns** moderately thin data | **Moderate confidence** | No or very minor concerns regarding coherence, minor concerns regarding methodological limitations, and moderate concerns regarding relevance and adequacy |
| ***Subtheme 2: Building relationships*** | | | | | | | |
| Finding 3: The animal, through intuition or training, may facilitate a therapeutic relationship with people with dementia | Fields, 2017; Fields et al., 2019; Kongable et al., 1990; Lassell et al., 2022a; Swall et al., 2015; Swall et al., 2017 | **Moderate** **concerns**  based on the assessments of 1 study (2 reports) with no or very minor, 2 (3 reports) with minor concerns and 1 with serious methodological limitations | **No or very minor concern** | **Moderate concerns**  regarding studies had moderate geographical spread  Intervention had limited AAI activities and animals  Participants included people living with dementia, service providers and family members.  Studies had a good time range | **Minor concerns** relatively rich data | **Moderate confidence** | No or very minor concerns regarding coherence, minor concerns regarding adequacy, and moderate concerns regarding methodological limitations and relevance |
| Finding 4: People with dementia may begin to feel a sense of protectiveness and responsibility for caring for the animals | Kawamura et al., 2009; Hodgson et al., 2022; Swall, 2015; Swall et al., 2015; Swall et al., 2017 | **Minor concerns** based on assessment of 1 study (3 reports) with no or very minor, 2 studies (2 reports) with minor methodological limitations | **No or very minor concerns** | **Moderate concerns**  regarding studies had moderate geographical spread  Participants included both people living with dementia and service providers.  Intervention had limited AAI animals and activities | **Minor concerns** | **Moderate confidence** | No or very minor concerns regarding coherence, minor concerns regarding methodological limitations and adequacy, and moderate concerns regarding relevance |
| Finding 5: Animals could be a catalyst for developing enhanced relationships between people with dementia and others through shared experiences with the animals | Fields, 2017; Fields et al., 2019; Hodgson et al., 2022; Kawamura et al., 2009; Lassell et al., 2022b; Kongable et al., 1990; Swall et al., 2016 | **Minor concerns** based on assessment of 1 study (1 report) with no or very minor, 4 (5 reports) with minor and 1 with serious methodological limitations | **Minor concerns** as a few contradictory data | **Minor concerns** regarding studies had moderate geographical spread  Participants included people living with dementia in moderate range of diagnosis and state, age, and gender  Participants included services providers and family members  Interventions inclusive of a range of animals  Studies had a good time range | **Minor concerns** as moderately rich data | **High confidence** | Minor concerns regarding methodological limitations, coherence, relevance, and adequacy |
| **Theme 2: Growing: Engaging in AAI** | | | | | | | |
| ***Subtheme 1: A rich experience*** | | | | | | | |
| Finding 6: Interactions in AAI could be a multidimensional experience from sensory input to physical and cognitive interactivity. | Busselman, 2017; Hodgson et al., 2022; Fields, 2017; Fields et al., 2019; Kongable et al., 1990; Lassell et al., 2022a; Lassell et al., 2022b; Swall, 2015; Swall et al., 2015; Swall et al., 2016; Swall et al., 2017; Swall et al., 2019 | **Minor concerns** based on assessment of 3 studies (5 reports) with no or very minor, 5 (6 reports) with minor and 1 with serious methodological limitations | **No or very minor concerns** | **Minor concerns** regarding studies had moderate geographical spread  Participants included people living with dementia in moderate range of diagnosis, state, age and gender  Participants included services providers and family members  Interventions inclusive of a range of animals  Studies had a good time range | **No or very minor concerns** regarding moderately rich data | **High confidence** | No or very minor concerns regarding coherence and adequacy, and minor concerns regarding methodological limitations and relevance |
| Finding 7: Interaction with animals could trigger memories, positive and negative, for people with dementia | Fields, 2017; Fields et al., 2019; Kongable et al., 1990; Kawamura et al., 2009; Lassell et al., 2022a; Swall, 2015; Swall et al., 2015; Swall et al., 2016; Swall et al., 2017 | **Minor concerns** based on assessment of 2 studies (4 reports) with no or very minor, 3 (4 reports) with minor and 1 with serious methodological limitations | **No or very minor concerns** | **Minor concerns** regarding studies had moderate geographical spread  Participants included people living with dementia in a good range of diagnosis, state, age, and gender  Participants included services providers and family members  Interventions inclusive of moderate range of animals  Studies had a good time range | **No or very minor concerns** regarding moderately rich data | **High confidence** | No or very minor concerns regarding coherence and adequacy, and minor concerns regarding methodological limitations and relevance |
| Finding 8: AAI provides opportunity for people with dementia to interact with others more socially and to connect with nature | Casey et al., 2018; Fields, 2017; Fields et al., 2019; Hodgson et al., 2022; Kongable et al., 1990; Kawamura et al., 2009; Lassell et al., 2022a; Lassell et al., 2022b; Swall, 2015; Swall et al., 2015; Swall et al., 2016; Swall et al., 2017; Swall et al., 2019 | **Minor concerns** based on assessment of 3 studies (5 reports) with no or very minor, 5 (6 reports) with minor, 1 with moderate and 1 with serious methodological limitations | **No or very minor concerns** | **Minor concerns** regarding studies had relatively good geographical spread  Participants included people living with dementia in a good range of diagnosis, state, age, and gender  Participants included services providers and family members  Interventions inclusive of a range of animals  Studies had a good time range | **No or very minor concerns** regarding moderately rich data | **High confidence** | No or very minor concerns regarding coherence and adequacy, and minor concerns regarding methodological limitations and relevance |
| ***Subtheme 2: The benefits of AAI*** | | | | | | | |
| Finding 9: People with dementia, engaging AAI, may experience improved mood, and physical improvements | Casey et al., 2018; Fields, 2017; Fields et al., 2019; Hodgson et al., 2022; Kongable et al., 1990; Kawamura et al., 2009; Lassell et al., 2022a; Lassell et al., 2022b; Swall, 2015; Swall et al., 2015; Swall et al., 2016; Swall et al., 2017; Swall et al., 2019 | **Minor concerns**  based on assessment of 3 study (5 reports) with no or very minor, 5 (6 reports) with minor, 1 with moderate and 1 with serious methodological limitations | **Minor concerns** regarding a few conflict data | **Minor concerns**  regarding studies had relatively good geographical spread, but all high-income countries  Participants included people living with dementia in a good range of diagnosis, state, age, and gender  Participants included services providers and family members  Intervention had a range of AAI animals and activities  Studies had a good time range | **No or very minor concerns** regarding moderately rich data | **High confidence** | No or very minor concerns regarding adequacy, and minor concerns regarding methodological limitations, coherence, and relevance |
| Finding 10: AAI for people with dementia may have positive effects on cognitive functions and social ability; and may reduce BPSD symptoms | Casey et al., 2018; Fields, 2017; Fields et al., 2019; Hodgson et al., 2022; Kongable et al., 1990; Kawamura et al., 2009; Lassell et al., 2022a; Swall et al., 2015; Swall et al., 2016; Swall et al., 2019 | **Minor concerns**  based on assessment of 3 studies (3 reports) with no or very minor, 4 (5 reports) with minor, 1 with moderate and 1 with serious methodological limitations | **Minor concerns** regarding minor conflict data | **Minor concerns** regarding studies had relatively good geographical spread  Participants included people living with dementia in a good range of diagnosis, state, age, and gender  Participants included services providers and family members  Intervention had a relatively good range of AAI animals and activities  Studies had a good time range | **No or very minor concerns** | **High confidence** | No or very minor concerns regarding adequacy, and minor concerns regarding methodological limitations, coherence, and relevance |
| Finding 11: Staff, volunteers and carers of people with dementia may observe very positive benefits of AAI for the person living with dementia; and reported a hope for the programmes to continue to see the long-term impact | Casey et al., 2018; Fields, 2017; Fields et al., 2019; Hodgson et al., 2022; Kongable et al., 1990; Lassell et al., 2022a; Lassell et al., 2022b; Swall, 2015; Swall et al., 2016; Swall et al., 2019 | **Minor concerns**  based on assessment of 2 studies (3 reports) with no or very minor, 4 (5 reports) with minor concerns, 1 with moderate and 1 with serious methodological limitations | **No or very minor** | **Minor concerns** regarding studies had moderate geographical spread  Participants included people living with dementia in a moderate range of diagnosis, state, age, and gender  Participants included services providers and family members  Intervention had a relatively good range of AAI animals and activities  Studies had a good time range | **Minor concerns** regarding comparably moderate thin data and superficial data | **High confidence** | No or very minor concerns regarding coherence, and minor concerns regarding methodological limitations, relevance, and adequacy |
| **Theme 3: Nurturing: Making AAI work** | | | | | | | |
| ***Subtheme 1: Individualised and holistic approach*** | | | | | | | |
| Finding 12: AAI needs to be a flexible and adaptive intervention providing various activities delivered tailored to individual needs. | Busselman, 2017; Casey et al., 2018; Fields, 2017; Fields et al., 2019; Hodgson et al., 2022; Kongable et al., 1990; Kawamura et al., 2009; Lassell et al., 2022a; Lassell et al., 2022b; Swall, 2015; Swall et al., 2016 | **Minor concerns**  based on assessment of 1 study (2 reports) with no or very minor, 6 (7 reports) with minor, 1 with moderate and 1 with serious methodological limitations | **No or very minor concerns** | **Minor concerns** regarding studies had relatively good geographical spread  Participants included people living with dementia in a moderate range of diagnosis, state, age, and gender  Participants included services providers and family members  Intervention had a relatively good range of AAI animals and activities  Studies had a good time range | **No or very minor concerns** regarding moderately rich data | **High confidence** | No or very minor concerns regarding coherence and adequacy, and minor concerns regarding methodological limitations and relevance |
| Finding 13: A multicomponent AAI programme can provide additional everyday activities and a rich holistic experience for people with dementia | Busselman, 2017; Fields, 2017; Fields et al., 2019; Hodgson et al., 2022; Lassell et al., 2022a; Lassell et al., 2022b | **Minor concerns**  based on assessment of 5 studies (6 reports) with minor methodological limitations | **No or very minor concerns** | **Moderate concerns** regarding studies had moderate geographical spread  Participants included people living with dementia in a limited range of diagnosis, state, age, and gender  Participants included services providers and family members  Intervention had a limited range of AAI animals and activities | **Minor concerns** regarding moderate thin data | **Moderate confidence** | No or very minor concerns regarding coherence, minor concerns regarding methodological limitations and adequacy, and moderated concerns regarding relevance |
| ***Subtheme 2: Training and support*** | | | | | | | |
| Finding 14: Experienced staff in AAI are valuable, conducting training around dementia care and AAI to improve staff’s knowledge and skills is necessary | Fields, 2017; Fields et al., 2019; Kongable et al., 1990 | **Moderate concerns**  based on assessment of 1 study (2 reports) with minor and 1 with serious methodological limitations | **No or very minor concerns** | **Moderate concerns** regarding studies had limited geographical spread  Participants included people living with dementia in a limited range of diagnosis, state, age, and gender  Participants included services providers  Intervention had a limited range of AAI animals and activities | **Moderate concerns** regarding moderate thin data | **Moderate confidence** | No or very concerns regarding coherence, and moderate concerns regarding methodological limitations, relevance, and adequacy |
| Finding 15: There may be associated risks for people with dementia engaging in AAI, which may be reduced through safety measures or supports from staff and care partners | Busselman, 2017; Fields, 2017; Fields et al., 2019; Hodgson et al., 2022; Kongable et al., 1990; Lassell et al., 2022b; Swall, 2015; Swall et al., 2016 | **Minor concerns**  based on assessment of 1 study (2 reports) with no or very minor, 4 (5 reports) with minor and 1 with serious methodological limitations | **No or very minor concerns** | **Moderate concerns** regarding studies had moderate geographical spread  Participants included people living with dementia in a limited range of diagnosis, state, age, and gender  Participants included services providers and family members  Intervention had a moderate range of AAI animals and activities | **Minor concerns** regarding moderately rich data | **High confidence** | No or very minor concerns regarding coherence, minor concerns regarding methodological limitations and adequacy, and moderate concerns regarding relevance |

**Supplementary File 5. Supporting Quotations**

| **Theme** | **Subtheme** | **Finding** | **Quotation** | **Reports contributing to the review finding** |
| --- | --- | --- | --- | --- |
| Planting: Connecting with animals | Willingness to connect | Finding 1: The willingness of people with dementia, care partners and staff to engage with AAI may depend on prior experience or their attitude towards animals in general | *‘…some participants took a keen interest in the hens…many older people who live in a care home demonstrated curiosity about the hens’* (Hodgson et al., 2022).  *‘Respondents who worked the day shift also described curiosity about the animals among staff who were not directly involved’* (Casey et al., 2018).  *‘Staff throughout the whole building were like Boh my goodness the animals are here…just take time out of their own day to come in and see how things were going’* (Casey et al., 2018).  *‘…maybe people who work with the residents on a daily basis see them in a different light or experience burnout and as a result do not want to always encourage engagement’* (Fields, 2017).  *‘All participants either liked dogs or were neutral in their feelings…’* (Kawamura et al., 2009).  *‘…Memory Lane staff noticed workers from other units of the institution and programs stopping by to see the animals and to watch the residents enjoy their time interacting with them…the staff would come down and specifically go out to see the animals’; ‘we always love to see an opportunity for our residents to enjoy something very special…we love to see in enrichment in our resident's lives’* (Casey et al., 2018). | Casey et al., 2018; Fields, 2017; Fields et al., 2019; Hodgson et al., 2022; Kawamura et al., 2009; Kongable et al., 1990; Lassell et al., 2022a; Lassell et al., 2022b; Swall et al., 2016 |
|  |  | Finding 2: Despite initial resistance, people with dementia may begin to engage with AAI after a few activities | *‘Coming initially, Opal (participant) was not getting into any of the activities during the program, ‘No. I don't want to wear a helmet. Thank you.’ But she did end up putting on her helmet to ride…. after she rode for about 10 minutes’* (Fields, 2017).  *‘…there were some that were reticent about touching them… And then, at the finish, she’s, ‘Come on, then, go and get the chicken.’ And she’s got a towel on her knee. ‘Give me it here.’ And it sits on her knee’* (Hodgson et al., 2022).  *‘…he was distressed by directing him to the henhouse. His behaviour changed and he stopped to calmly watch the hens’* (Hodgson et al., 2022). | Fields, 2017; Hodgson et al., 2022 |
|  | Building relationships | Finding 3: The animals, through intuition or training, may facilitate a therapeutic relationship with people with dementia | *‘I have observed the horses during the program-they tune into our folks with dementia in a different way. I have a horse that may be naughty in some situations but once they [horse] get around the elderly they seem to sense that they [elderly] are not a threat. So, they are just calmer and that helps the residents relax’* (Fields, 2017; Fields et al., 2019).  *‘Through obedience training, the dog learned to pick up on cues of aggressive behaviour, thereby minimizing the potential for injury’* (Kongable et al., 1990).  *‘…horse Rocket would provide an immediate response…D described this connection as a ‘bond’, which was also shaped by seeing his wife C’s positive connection with Rocket’* (Lassell et al., 2022a).  *‘They showed deep, tender feelings towards the dog, while at the same time it seemed like the dog understood the person’s limitations’* (Swall et al., 2015). | Fields, 2017; Fields et al., 2019; Kongable et al., 1990; Lassell et al., 2022a; Swall et al., 2015; Swall et al., 2017 |
|  |  | Finding 4: People with dementia may begin to feel a sense of responsibility for caring for the animals | *‘They expressed feelings of ownership and talked with the confidence of someone very familiar with the dogs…Momo [the dog] … I knew she would settle down in about 10 minutes. Once she settles down … then I understand, ‘Momo is fine’* (Kawamura et al., 2009).  *‘On a night-time he says “Oh, make sure the chickens are locked in”’* (Hodgson et al., 2022).  *‘Mr. Edgar: Are you limping, my friend? [He looks at the…dog.] Dog handler (DH): Yeah, he has a bit of pain in his front end. Mr Edgar: OK . . .. [He follows the dog with his eyes. His voice becomes somewhat quieter and darker, and his expression becomes serious.] DH: Do you think it will pass? Mr Edgar: Yes, I hope so … DH: yes … I think so. Mr Edgar: Yeah, let’s hope so.* *DH: He gets medicine for his limping. Mr Edgar: Ooh well. DH: So, so the vet thinks so. Mr Edgar: Yes, yes it appears that he has problems when he walks … Oh, oh, oh poor little chap, yes, yes’* (Swall et al., 2017). | Kawamura et al., 2009; Hodgson et al., 2022; Swall, 2015; Swall et al., 2015; Swall et al., 2017 |
|  |  | Finding 5: Animal could be a catalyst for developing enhanced relationships between people with dementia and others through shared experiences with the animals | *‘Creating connections are huge-with the horse, with other people. Just being able to have a shared experience that is going on with everyone else around you’* (Fields et al., 2019).  *‘A positive moment for me was when two female residents were sitting and chatting and I heard them say ‘lovely chicken, I love you.’ It was wonderful to hear. They had just moved into the home and they didn’t know each other. They made a connection together with this hen and that was the start of their friendship. It was wonderful’* (Hodgson et al., 2022).  *‘It’s like I get to go to church and be affirmed, but without any overt words. . .I felt very empowered like I’m doing a good thing and I’m close to other people that believe the same way’* (Lassell et al., 2022b).  *‘It helps to see other people who are going through the same thing. I’m not the only one going through this and neither is P [patient]’* (Lassell et al., 2022b). | Fields, 2017; Fields et al., 2019; Hodgson et al., 2022; Kawamura et al., 2009; Lassell et al., 2022b; Kongable et al., 1990; Swall et al., 2016 |
| Growing: Engaging in AAI | A rich experience | Finding 6: Interactions in AAI could be a multidimensional experience ranging from sensory input to physical and cognitive interactivity | *‘It can be a really positive experience for people with dementia-touching them [horses], smelling them, hearing their sounds-they really seem to stimulate so much which can be a positive thing’* (Fields, 2017; Fields et al., 2019).  *‘They get involved. Like, when the lady sits down and they’re talking about the names of the chickens. And then telling them a story, and they’re interacting with the story. They might sing a song about the hens’* (Hodgson et al., 2022).  *‘When we’re grooming him [horse] or when she’s riding him, it’s just this continual communication and most of its nonverbal’* (Lassell et al., 2022a).  *‘I put him (the dog) on the person’s bed so he was close to the*  *man. He was sleeping with his arm around the dog close, and he did not see him and did not know he was there, but just felt the dog. And it was like pressing a button, the breathing slows down completely with calm breathing, completely quiet breathing’* (Swall et al., 2016). | Busselman, 2017; Hodgson et al., 2022; Fields, 2017; Fields et al., 2019; Kongable et al., 1990; Lassell et al., 2022a; Lassell et al., 2022b; Swall, 2015; Swall et al., 2015; Swall et al., 2016; Swall et al., 2017; Swall et al., 2019 |
|  |  | Finding 7: Interaction with animals could trigger memories, positive and negative, for people with dementia | *‘…some of the residents that come have backgrounds with farms or were ranchers. While they are at Hearts and Horses, it really gives them those memories that maybe they couldn’t remember until they were exposed to it again’* (Fields, 2017; Fields et al., 2019).  *‘When I entered the brain surgery unit in the hospital, I asked*  *my family to bring my dog to see me. They did, and I could see*  *my dog from the window on the eighth floor of the hospital…. My daughter took the dog to the opposite side of the road, but the dog didn’t really look up …’* (Kawamura et al., 2009).  *‘Mrs. David : “Yes, yes … Sally, I had a dog named that … Yeah … it’s a long time ago now”. Still looks down towards the ﬂoor, looks up and sees the windows, and does not smile, bends her head down again and looks down at the ﬂoor’* (Swall et al., 2015). | Fields, 2017; Fields et al., 2019; Kongable et al., 1990; Kawamura et al., 2009; Lassell et al., 2022a; Swall, 2015; Swall et al., 2015; Swall et al., 2016; Swall et al., 2017 |
|  |  | Finding 8: AAI provides opportunity for people with dementia to interact with others more socially and to connect with nature connection | *‘It’s like it’s part of the family and they’re...It’s a big conversation starter as well. Like, when the first egg was laid, it was like, ‘Whoah... ‘It was like the crown jewels. [Name] was walking around and showing everybody...’* (Hodgson et al., 2022).  *‘... many talk about their own future death ... a man with aphasia, he talked to the dog when he was approaching the end of his life: “Soon, I won’t be here anymore” ... and he did not look at me; he was talking to the dog the whole time …’* (Swall et al., 2019).  *‘they (volunteers) are all young people, so I like getting energy from them. They remind me of my younger days, and I remember that I was once like that a long time ago’* (Kawamura et al., 2009).  *‘I think there are so many things. One being outside and having this connection with nature’* (Fields 2017; Fields et al., 2019).  *‘We have definitely brought the outside in…And the reason we’ve chosen that area is specifically for those residents who do not want to come downstairs. Or do not want to go outside. So we’re going to create an inside garden…so, they will see directly where the chickens are’* (Hodgson et al., 2022). | Casey et al., 2018; Fields, 2017; Fields et al., 2019; Hodgson et al., 2022; Kongable et al., 1990; Kawamura et al., 2009; Lassell et al., 2022a; Lassell et al., 2022b; Swall, 2015; Swall et al., 2015; Swall et al., 2016; Swall et al., 2017; Swall et al., 2019 |
|  | The benefits of AAI | Finding 9: People with dementia, engaging AAI, may experience improved mood, and physical improvements | *‘…that it's making a different in the resident at that time… and I will keep repeating it that it was a good thing for the home, for the residents for the staff. I seen the residents are more uplifting, smiling, it was very effective, especially with the residents who are more withdrawn. They were light, they were lit up’* (Casey et al., 2018).  *‘Many of the residents really enjoy the trip to the ranch [EAAP]’* (Fields, 2017; Fields et al., 2019).  *‘When she [P] gets up and walks, she does stand up straighter’, ‘couldn’t even make it to the car’ without taking a break and now she was able to continuously walk her horse to the pasture after riding’* (Lassell et al., 2022a).  *‘He usually wouldn’t do anything. We’re having to assist him with his meals and he can’t walk anymore since he fractured his hip. But as soon as I said, “Come on, you need to show me what colours go where”, He was away’* (Hodgson et al. 2022). | Casey et al., 2018; Fields, 2017; Fields et al., 2019; Hodgson et al., 2022; Kongable et al., 1990; Kawamura et al., 2009; Lassell et al., 2022a; Lassell et al., 2022b; Swall, 2015, Swall et al., 2015; Swall et al., 2016; Swall et al., 2017; Swall et al., 2019 |
|  |  | Finding 10: AAI for people with dementia may have positive effects on cognitive functions and social ability; and may have reduced BPSD symptoms | *‘I was shocked about some of the residents that wander and pace up and down the halls, sometimes like for an hour straight, could actually sit and enjoy and just be involved in. I found that they would sit for the whole thing, which I thought was great’* (Casey et al., 2018).  *‘They [residents] seem to remember the steps of mounting and dismounting, even though we help them each time with the steps. Oh, I’m [resident] to take my feet out of the stirrups, then lift my leg across the horse. Each time we see that they get a little more confident and comfortable with the process’* (Fields, 2017; Fields et al., 2019).  *‘Whether it’s getting a ring or a beanbag [at RMTM] or making a decision about going to a restaurant and ordering food that’s really changed a lot’* (Lassell et al., 2022a).  *‘They enjoy touching, stroking and feeling the animals. It relieves the tension and stress. It can be calming. It’s surprising what a difference it makes – when someone is distressed or agitated it helps to calm them’* (Hodgson et al., 2022)  ‘*Gertrude (participant) was just speaking clearly; she was singing and all the words together were making sense. It was an improvement from her typical gurgled and soft speech*’ (Fields, 2017; Fields et al., 2019).  *‘Before [adaptive riding] it was more “yes” or “no” answers and even then she [mother] wouldn’t really elaborate and now she’s interjecting into conversations and saying what she’s thinking and [expressing] her point of view’* (Lassell et al., 2022a). | Casey et al., 2018; Fields, 2017; Fields et al., 2019; Hodgson et al., 2022; Kongable et al., 1990; Kawamura et al., 2009; Lassell et al., 2022a; Swall et al., 2015, Swall et al., 2016; Swall et al., 2019 |
|  |  | Finding 11: Staff, volunteers and carers of people with dementia may observe very positive benefits of AAI for the person living with dementia; and reported a hope for the programmes to continue to see the long-term impact | *‘When I [daughter] looked at mom just a bit ago, I thought, this is it; it is the end of her life. Then you get these incredible pictures of her excitement and hear that your mom who is nonverbal makes so many connections in the program, it is just an amazing feeling’* (Fields, 2017; Fields et al., 2019).  *‘Thank you very much. You’ve done something that nobody else has done. You’ve made mum laugh. She’s seeing you lot running around and chasing the hens’* (Hodgson et al., 2022).  *‘And hopefully that will have some long-term lasting impact on wellbeing’* (Hodgson et al., 2022). | Casey et al., 2018; Fields, 2017; Fields et al., 2019; Hodgson et al., 2022; Kongable et al., 1990; Lassell et al., 2022a; Lassell et al., 2022b; Swall, 2015; Swall et al., 2016; Swall et al., 2019 |
| Nurturing: Making AAI work | Individualised and holistic approach | Finding 12: AAI needs to be a flexible and adaptive intervention providing various activities delivered tailored to individual needs | *‘…we [staff] need to know which people like to participate in music versus storytelling, jokes, or gardening. Everybody has their preferences and we pride ourselves in knowing our residents [people with dementia] and allowing them to decide what they want’* (Fields, 2017).  *‘Some residents don’t go outside, or leave their bedrooms. I’ve got one resident that does like me to bring the hen into her room. I bring a different one each time, and the hen sits on her table and she has a bit chat with it. She talks to it, talks about its eyes and stuff like that’* (Hodgson et al., 2022). | Busselman, 2017; Casey et al., 2018; Fields, 2017; Fields et al., 2019; Hodgson et al., 2022; Kongable et al., 1990; Kawamura et al., 2009; Lassell et al., 2022a; Lassell et al., 2022b; Swall, 2015; Swall et al., 2016 |
|  |  | Finding 13: A multicomponent AAI programme can provide additional everyday activities and a rich holistic experience for people with dementia | *‘The program is an outing for the residents so that is one element; it involves an animal as another layer; and then there is the movement and socialization piece of the program. I think having this kind of recipe- those things together, I think are what makes the program so successful. I think if you took the elements apart individually you would not have that rich experience’* (Fields et al., 2019).  *‘It’s been nice to have different materials for residents to work with. You know, they have produced some fantastic work. There’s quite a lot of them point out that “I did that”’* (Hodgson et al., 2022).  *Which has been really nice, it’s the ownership that they’ve got over something they produce which has been really good’* (Hodgson et al., 2022). | Busselman, 2017; Fields, 2017; Fields et al., 2019; Hodgson et al., 2022; Lassell et al., 2022a; Lassell et al., 2022b |
|  | Training and support | Finding 14: Experienced staff in AAI are valuable, conducting training around dementia care and AAI to improve staff’s knowledge and skills is necessary | *‘Consistency is really important with staff and with volunteers. I think a more extensive training day about dementia, the residents, and the role of the program for our next session needs to happen. I want everyone to feel like they are part of a team’* (Fields, 2017).  *‘I would also like to come up with some kind of really simple thing that tracks progress or changes over time. Those are goals of mine, we do track outcomes with other programs here but not with this one [RM]. I feel like there is definitely room for improvement with that on our end.’* (Fields, 2017). | Fields, 2017; Fields et al., 2019; Kongable et al., 1990 |
|  |  | Finding 15: There may be associated risks for people with dementia engaging in AAI, which may be reduced through safety measures or supports from staff and care partners | *‘I always hear from people in the community how surprised they are to learn that people with dementia are actually involved in programming at Hearts and Horses; they often think there are more risks than rewards for this population like falling or easily getting confused and anxious’* (Fields, 2017; Fields et al., 2019).  *‘Yes, horses can be dangerous but we do everything we can to mitigate risk with our side walkers, horse leaders, the horses we have selected for the program, as well as matching residents to activities. I think the more people that find out about our program and how successful it is safety wise, more programs like ours will start to pop up.’* (Fields, 2017; Fields et al., 2019).  *‘That’s one of the things that we’re having to look at. Is the safety. So, we are looking at maybe taking the box away and making it a bigger area. And then having a nice seating area, so the residents can watch the hens more’* (Hodgson et al., 2022).  *‘It made the experience much more enjoyable not just for me but for her to know that I’m right there the whole time, and talking, and being with her’* (Lassell et al., 2022b).  *‘But, I just felt better that I could see more up close what was going on and ways to make her [P] feel more comfortable while she was riding Raymond [donkey]’* (Lassell et al., 2022b). | Busselman, 2017; Fields, 2017; Fields et al., 2019; Hodgson et al., 2022; Kongable et al., 1990; Lassell et al., 2022b; Swall, 2015; Swall et al., 2016 |
